# Executive Function Among Older Adults With Bipolar Disorder: A GAGE-BD Analysis

**DOI:** 10.1016/j.jagp.2025.11.017

**Published:** 2025-12-07

**Authors:** Federica Klaus, Erin Appiah, Izabela G. Barbosa, Peijun Chen, Ariel Gildengers, Paula Villela Nunes, Farren Briggs, Nicole Fiorelli, Ashley N. Sutherland, James E. Emanuel, Paola Lavin, Benoit H. Mulsant, Melis Orhan, Regan Patrick, Tarek K. Rajji, Sigfried Schouws, Soham Rej, Annemiek Dols, Martha Sajatovic, Lisa T. Eyler

**Affiliations:** Department of Psychiatry (FK, ANS, LTE), University of California San Diego, San Diego, CA; School of Medicine (EA), University of California San Diego, San Diego, CA; Departamento de Psiquiatria (IGB), Faculdade de Medicina, Universidade Federal de Minas Gerais, Belo Horizonte, Brazil; Department of Psychiatry (PC), University Hospitals Cleveland Medical Center, Case Western Reserve University School of Medicine, Cleveland, OH; Geriatric Research, Education and Clinical Center (GRECC) and Psychiatric Service (PC), VA Northeast Ohio Healthcare System, Cleveland, OH; Department of Psychiatry (AG, JEE), University of Pittsburgh School of Medicine, Western Psychiatric Hospital; Department of Psychiatry (PVN), University of São Paulo Medical School, São Paulo, Brazil; University of British Columbia (PVN), Vancouver, Canada; Department of Public Health Sciences (FB), Miller School of Medicine, University of Miami, Miami, FL; Case Western Reserve University School of Medicine (MS) (NF), University Hospitals Cleveland Medical Center, Cleveland, OH; Department of Psychiatry (PL), Lady Davis Institute, Montreal, QC, Canada; Department of Psychiatry (BHM), Temerty Faculty of Medicine, University of Toronto, Toronto, Canada; Centre for Addiction and Mental Health, Toronto, Canada; The Royal (BHM), Ottawa, Canada; Institute of Clinical Psychology (MO), Leiden University, Leiden, Netherlands; Departments of Neuropsychology & Geriatric Psychiatry (RP), McLean Hospital, Belmont MA; Department of Psychiatry (TKR), O’Donnell Brain Institute, University of Texas Southwestern Medical Center, Dallas, TX; GZZ inGeest Specialized Mental Health Care (SS), Department of old age psychiatry, Amsterdam, the Netherlands; Department of Psychiatry (SR), Jewish General Hospital/Lady Davis Institute, McGill University, Montreal, Canada; School of Nursing and Midwifery (SR), Queen’s University Belfast, Belfast, UK; Department of Psychiatry (AD), Brain Division, UMC Utrecht, Utrecht University, Utrecht, Netherlands; and the University Hospitals Cleveland Medical Center (MS), Case Western Reserve University School of Medicine, Cleveland, OH.

**Keywords:** Executive function, bipolar disorder, older adults, functioning

## Abstract

**Objectives:**

Executive function deficits in bipolar disorder (BD) are major contributors to disability in older age BD (OABD). We investigated the difference between OABD and age-equated healthy comparators (HC); and, in the OABD group, the associations of executive function with age, symptom severity, global cognition, and daily functioning.

**Design:**

Cross-sectional analysis of executive function in OABD versus HC.

**Setting:**

Analysis of large archival dataset harmonized from 12 international OABD studies.

**Participants:**

*Older adults (≥50 years) with OABD* (n = *614) and HC* (n = *192)*.

**Measurements:**

Executive function was assessed via Trail Making Test B (TMT-B) completion time; covariates included age, self-reported gender, education, study site, medications (antipsychotics, lithium), and psychomotor speed.

**Results:**

Executive function was worse in OABD than in HC, even after controlling for psychomotor speed (p < 0.001). In the OABD group, test completion was associated with less severe manic symptoms (p < 0.001). Worse executive function was associated with older age (p = 0.001), antipsychotic use (p < 0.001), worse global cognition (p < 0.001), and worse daily functioning (p < 0.001).

**Conclusions:**

Executive dysfunction is a prominent feature of OABD, associated with several demographic and clinical characteristics. Future longitudinal studies of executive function and OABD need to assess the individual impact of impairment in executive function on everyday functioning to inform personalized interventions targeting specific patient subgroups.

## OBJECTIVE

Older adults with bipolar disorder (OABD) will constitute more than half of the bipolar disorder (BD) population within the next 10 years,^1^ but research is sparse about the aging process in BD, specifically cognitive impairment as a key burden. The evolution of symptoms and everyday functioning across the life span is poorly comprehended.^2^

The majority of research in BD has been conducted on younger individuals (<50 years) and does not necessarily elucidate how BD affects cognitive impairment in addition to the influence of normal aging processes. Among the most persistent symptoms are cognitive deficits, especially in attention, verbal learning, and executive function, present in 40% to 50% of individuals with OABD in the euthymic phase.^3-7^ While OABD performed worse than a healthy comparison (HC) group on cognitive tests in a longitudinal study, the rate of cognitive decline over 5 years was not significantly different.^8^ Learning, memory, and processing speed present large effect sizes of impairment, whereas language and visuoconstruction did not reveal significant differences between OABD and HC.^9^ A cross-sectional study using pooled, harmonized data from the Global Aging and Geriatric Experiments in Bipolar Disorder (GAGE-BD^2^) consortium found no difference in global cognition between OABD and HC.^10^ In line with meta-analytic findings on individual cognitive domains, including 328 euthymic OABD cases and 302 HC,^9^ this suggests that differences in cognitive performance may lie in individual domains rather than global cognition. The evaluation of a global cognition score might mask differences, highlighting the importance of investigating individual domains, such as executive function, which constitutes an essential skill for navigating everyday life in BD.^11^ These cognitive deficits impact clinical and functional outcomes, quality of life,^12-14^ and contribute to disability in OABD.^15^ It is hypothesized that cognition in BD worsens with disease progression^14^ tied to severe mood episodes.^16^

Better support of aging bipolar individuals requires insight into how cognitive performance in older age is affected. Findings on the relationship between disease course and cognition are mixed,^8,17,18^ pointing to the need for further investigation, considering taking into account aging effects and using pooled data to increase generalizability and power.

The objectives of this study were to investigate 1) the difference in executive function between OABD and HC and 2) among OABD, the associations of executive function with age, symptom severity, medication use (antipsychotics, lithium), global cognition, and functioning.

## METHODS

### Study Population

This is an analysis of data from a large archival set of baseline, cross-sectional, observational data on adults with BD and HCs, the GAGE-BD project, which includes pooled and harmonized international data from >1,300 individuals with BD.^2^ For the current analyses, data available as of May 2025 were used. Detailed information, sample characteristics, and metadata of contributing studies can be found elsewhere.^2,19^ Participants were included in the current analysis if they were aged ≥50 years and if data on executive function was available. The term OABD refers to individuals diagnosed as having BD who are ages 50 and older. This age cutoff is recommended by the International Society for BD (ISBD) Task Force on Older-Age Bipolar Disorder^20^ and is motivated by the fact that individuals with serious mental illness, such as BD, have a reduced life expectancy of 10 −20 years,^21^ and their biological age may precede their chronological age.^22,23^

Twelve studies from 7 sites contributed data of which 5 sites provided HC data. Approval to contribute data or determination of institutional review board (IRB) oversight exemption was obtained by each site’s IRB or ethics committees and by the GAGE-BD coordinating center (Case Western Reserve University School of Medicine, Cleveland, Ohio). Table S1 shows study site information, and Table S2 s inclusion/exclusion criteria.

### Sociodemographic and Clinical Characteristics

Demographic variables (age, gender, education level, employment status) and clinical variables (depression and manic severity, antipsychotic use due to its association with cognitive impairment,^24^ and lithium use due to its pro-cognitive, in addition to mood-stabilizing, effects, and age of onset) were harmonized across studies.^19^ Several studies did not include data on employment status; therefore, statistical models were run with and without employment status, and results were compared. In all studies, current mania symptom severity was measured with the Young Mania Rating Scale (YMRS).^25^ As current depressive symptoms were measured with the Hamilton Depression Rating Scale (HAM–D),^26^ Montgomery–Asberg Depression Rating Scale (MADRS),^27^ or the Center for Epidemiologic Studies Depression Scale (CES–D)^28^ in different study samples, data were transformed into one categorical depression severity variable with 3 categories: 0 = No depression (HAM-D ≤ 7; MADRS ≤6; CES- D ≤ 15), 1 = Mild or moderate depression (HAM-D 8–23; MADRS 7–34; CES-D 16–27), and 2 = Severe depression (HAM-D ≥ 24; MADRS ≥35; CES-D ≥ 28),^29^ General functioning was measured with the continuous Global Assessment of Functioning (GAF) scale, ranging from 0 to 100 (best functioning).^30^ Global cognition was assessed using a g-score, a harmonized measure based on available cognitive data for each participant, indicating general cognitive ability.^10^ In brief, for each participant, a z-score was calculated for their included test scores and these were entered into an unrotated principal component analysis (PCA) which was performed on a group and study-by-study basis. The first factor of the PCA was then used to calculate a factor score for each individual—their cognitive g-score. A negative g-score represents cognitive performance worse than average, whereas a positive g-score indicates cognitive performance above average.^10,31^

### Executive Function

Executive function was assessed using the Trail Making Test (TMT). The original TMT has 2 parts, A and B.^32^ TMT-A requires the participant to draw lines to connect encircled numbers (1 through 25) randomly distributed on a page in increasing order, as quickly as possible. In TMT-B, numbers (in increasing order) and letters (in alphabetical order) alternate. The most commonly used protocol was established by Reitan^33^ and requires the examiner to point out errors and direct the participant to self-correct. TMT-A is a measure of visual attention and psychomotor speed. TMT-B is a more complex measure of executive function, capturing different processes associated with performance, including inhibitory control, working memory, and attention. The main outcome measure is time to complete the test, which includes correction of errors. If the participant cannot complete the test in 5 minutes (300 sec), it is stopped, indicating cognitive impairment, such as mild cognitive impairment or dementia.^34^

The Delis-Kaplan Executive Systems (DKEFS)^35^ adaptation is a modified version with 5 trials: (1) Visual Scanning, (2) Number Sequencing, (3) Letter Sequencing, (4) Number-Letter Switching, and (5) Motor Speed. The 4 baseline trials (1, 2, 3, and 5) measure the requisite subskills (visual scanning, number sequencing, letter sequencing, and motor speed) needed to perform Trial 4, which is analogous to TMT-B.^32^

### Statistical Analysis

We investigated the following hypotheses (H):

1. Executive function performance will be worse in OABD compared to HC and this difference will be larger in the oldest group.
2. Within OABD, older age will be associated with worse executive function assessed by:
  - test completion versus non-completion and by;
  - test performance among those who completed the cognitive test.

Further, within OABD,

- more severe current clinical symptoms (more mania, more depression), earlier age of onset and use of antipsychotics, will be associated with poorer executive functioning and
- current lithium use will be associated with better executive function (test performance among those who completed the cognitive test and test completion versus non-completion).
- poorer executive function will be associated with poorer daily functioning.
- the relationship of poorer executive function and daily functioning will persist even when controlling for clinical characteristics.

Datasets were split into participants who completed the TMT-B test (raw test completion time <300 sec, OABD and HC) and those who did not (raw test completion time >300 sec, OABD only). Shapiro Wilk test examined distributional characteristics. Descriptive statistics were conducted using t-tests, chi-square (χ^2^), and Mann-Whitney U tests as appropriate. Dependent variables were transformed if needed to meet normality assumptions using an inverse rank normal score transformation with the Rankit method^36^ (applicable to GAF, g-score and executive function). Linear mixed models were used with study as a random effect and age, education and gender as fixed effects covariates in all models. Age and education were included due to general relation to cognition^32^ and gender was included because the ratio of men to women differed between HC and BDs. Secondary analysis included TMT-A performance (raw completion times in seconds) as additional covariate to the above models to isolate the executive function versus psychomotor speed aspects. Due to group differences (BD versus HC) in employment status, exploratory analyses included employment status, reducing sample size due to less availability of employment data.

The estimate of variance used was the standard deviation (SD). A 2-sided alpha of 0.05 was considered statistically significant. In the case of significant main effects, post hoc testing used the Least Significant Difference (LSD). Estimates of effect sizes for one-sample t-tests are given as Cohen’s d and for linear mixed models as partial eta squared (partial *η*^2^). Because of the lack of clarity in the field on how to calculate effect sizes in linear mixed models, partial *η*^2^ was calculated using general linear models with the same parameters as the linear mixed models and study as an additional fixed effect covariate (instead of a random effect). The results did not differ between the 2 model approaches unless mentioned.

Linear mixed models analyzed executive function differences between diagnostic groups (OABD versus HC) and the moderating role of age. Within the OABD group, models assessed the impact of age and clinical factors on test completion and performance. Additional models evaluated how executive function related to daily functioning and global cognition, retaining significantly associated clinical variables. See supplementary materials for detailed description.

In our sample, different TMT test versions were used at different sites, with the raw completion times in seconds for TMT-B and for the letter-number switching condition for DKEFS as the main outcome variable for executive function, based on the absence of statistical differences between average completion times between the 2 tests in OABD or HC, respectively. To control for psychomotor speed, we included in secondary analyses the raw completion times in seconds for TMT-A as covariate, which resulted in a smaller sample size.

## RESULTS

### Descriptive Statistics

**FIGURE 1.**
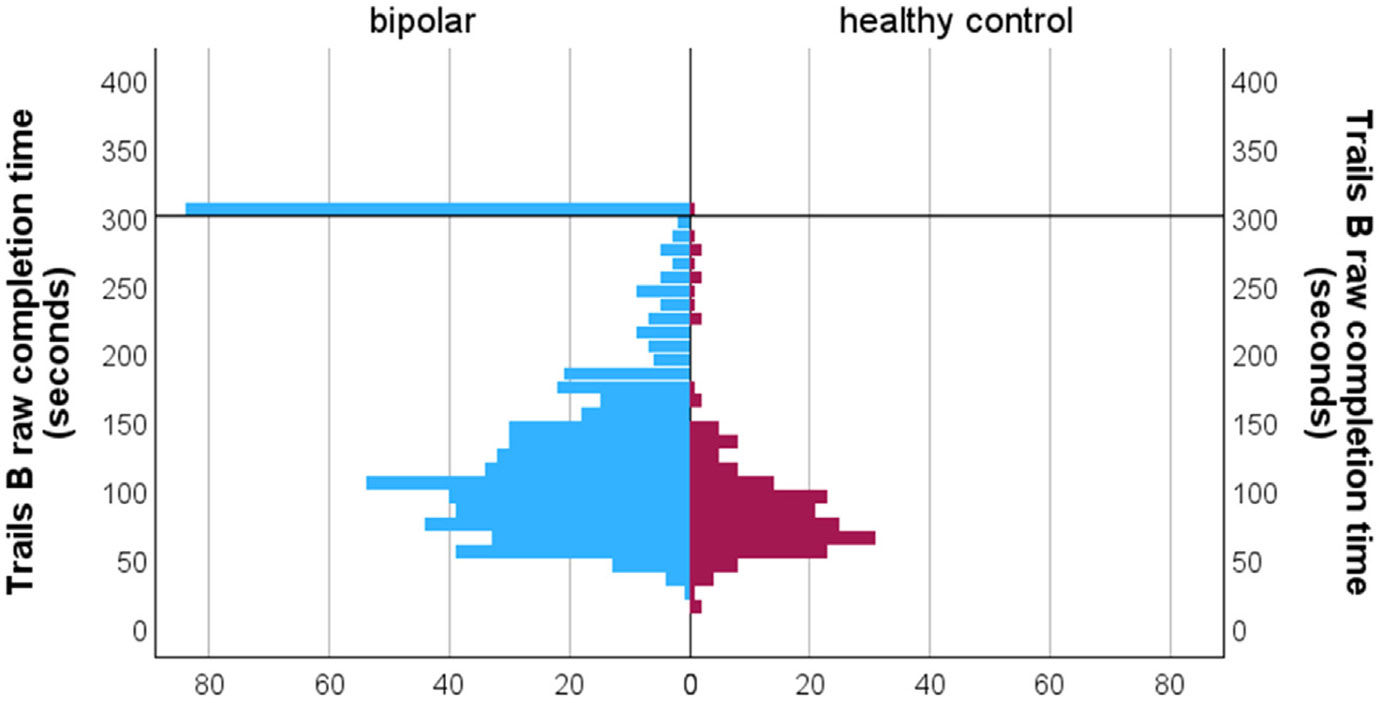
Frequency of TMT-B completion times (seconds) in bipolar disorder and healthy comparison participants. Higher completion times indicate worse cognitive performance. Black line indicates cut-off between test completers and non-completers (test completion time >300 sec).

In total, 614 OABD individuals and 192 HC aged 50 or older were analyzed. One HC and 84 OABD participants did not complete the TMT-B test (Table 1, Fig. 1).

Most OABD participants were not highly symptomatic. The majority (95%) had no to moderate severity of depression, and 64.8% had no mania (YMRS ≤ 12). Within OABD, the mean age was 66.5 years (SD: 7.6), 53% were female, the mean age of disease onset was 32.8 years (SD 16.6), participants had on average 13.7 years of education (SD: 7.6), 21% were working, average GAF was 54.1 (SD: 15.9) and 34% were current lithium and 34% current antipsychotics users.

Between OABD participants who completed the TMT-B test and those who did not, non-completers were older, were less likely to be employed and had fewer years of education, while gender did not differ (Table S3).

See supplementary Tables S4-S6 for model and result summaries.

### Executive Function

#### Group comparison of executive function as primary outcome (H1)

Within all participants who completed the TMT-B test, while controlling for study, age, education and gender, executive function was significantly worse in participants with OABD compared to HC (F(1,105) = 49.5, p < 0.001, partial *η*^2^ = 0.05; Fig. 1), with OABD having 0.53 seconds (95% Confidence interval [−0.722, −0.342]) slower TMT-B performance than HC. There was no significant interaction between diagnostic group and age (F(1, 650) = 1.33, p = 0.25, partial *η*^2^ = 0.001).

Upon inclusion of TMT-A performance to control for psychomotor speed in the above model, executive performance in BD remained significantly worse compared to HC (F(1,353) = 13, p < 0.001, partial *η*^2^ = 0.02) and there was still no significant interaction between diagnostic group and age (F(1, 556) = 0.1, p = 0.76, partial *η*^2^ < 0.001).

Adding employment status (*n* = 493) to all above models did not change the results except for the difference between OABD and HC becoming non-significant in the model that also included TMT-A (F (1,157) = 0.69, p = 0.41, partial *η*^2^ = 0.001) (see Table 2 for model overview and results).

#### Relationship of age with executive function among OABD (H2a/b)

Within OABD participants, when controlling for study, education and gender, there was a significant association of older age with worse executive function assessed by test completion status, i.e., TMT-B test completion was significantly associated with younger age (F(1,573) = 10.7, p = 0.001, partial *η*^2^ =0.02) and assessed by TMT-B test completion times within OABD test completers (F(1,295) = 76, p = <0.001, partial *η*^2^ = 0.11).

Upon inclusion of TMT-A performance in the model, age was not associated with test completion anymore in both models with (F(1,355) = 0, p = 0.98, partial *η*^2^ <0.001) and without inclusion of employment status (F (1,530) = 0.25, p = 0.62, partial *η*^2^ = 0.001). (see Table 3 with overview of models and results).

#### Variables associated with executive function (test completion time) among OABD test completers (H2c)

Within OABD TMT-B test completers, when controlling for study, age, education and gender, there was no statistically-significant association of executive function with depression severity (F (2,287) = 0.7, p = 0.5, partial *η*^2^ = 0.004), age of onset (F(1, 450) = 0.22, p = 0.64, partial *η*^2^ <0.001), current lithium use (F(1,471) = 1.2, p = 0.27, partial *η*^2^ < 0.001), or manic symptom severity (F(1,3) = 5.1, p = 0.11., partial *η*^2^ = 0.008). Better executive functioning was associated with no current antipsychotic medication use (F(1, 431) = 15, p < 0.001, FDR adj. p = 0.005, partial *η*^2^ = 0.04).

Upon inclusion of TMT-A performance in the above models, a significant association of fewer mania symptoms with better executive function emerged, however the effect size estimate from GLM was small and non-significant in this sample with relatively low severity of mania (F(1, 448) = 21.5, p < 0.001, FDR adj. p = 0.005, partial *η*^2^ = 0.006). The association with no antipsychotic use remained significant (F(1, 417) = 7.2, p = 0.008, FDR adj. p = 0.002, partial *η*^2^ = 0.023).

Upon addition of employment in the above models, changes were: no association of executive functioning with mania severity (F(1,3.9) = 6.6, p = 0.07, partial *η*^2^ = 0.008) and antipsychotic use (F(1,269) = 1.9, p = 0.16, partial *η*^2^ = 0.011) in the model including TMT-A.

#### Correlates of executive function (test completion) among OABD (H2d)

Within OABD participants, while controlling for study, age, education, and gender, TMT-B test completion was significantly associated with lower manic symptom severity (F(1,560) = 12.9, p < 0.001, FDR adj. p = 0.006, partial *η*^2^ = 0.022).

There was no significant association with age of onset (F(1,537) = 0.64, p = 0.43, partial *η*^2^ = 0.001), working (F(1,383) = 1.5, p = 0.22, partial *η*^2^ = 0.003), depression severity (F(1,546) = 0.13, p = 0.72, partial *η*^2^ < 0.001), current lithium use (F(1,537) = 0.31, p = 0.58, partial *η*^2^ = 0.001) or current antipsychotic medication use (F(1,538) = 2.5, p = 0.11, partial *η*^2^ = 0.005).

Results remained unchanged if TMT-A and/or employment status were included in the models.

#### Executive function as a variable associated with functioning and global cognition among OABD test completers (H2e/f)

Within OABD TMT-B test completers, while controlling for study, age, education and gender in a model that included also antipsychotic medication use due to the observed associations with executive function, a significant association of better executive function with better functioning was observed (F (1,290) = 15.1, p < 0.001, partial *η*^2^ = 0.049), and similar results were observed when not including antipsychotic use in the model (F(1,323) = 16.7, p < 0.001, partial *η*^2^ = 0.048).

A significant association of better executive function with global cognition was observed (F (1,392) = 238, p < 0.001, partial *η*^2^ = 0.396), and similar results were observed when not including antipsychotic use in the model (F(1,397) = 259, p < 0.001, partial *η*^2^ = 0.379).

Results remained unchanged if TMT-A and/or employment status were included in the models.

## DISCUSSION

Our overall objective was to contribute to improved diagnostic and treatment approaches focusing on executive function for OABD by understanding clinical factors that affect executive function, and its relationship with daily functioning and global cognition in OABD.

### Executive Function Between Older Adults With and Without BD

In line with previous literature,^9^ we found that HC outperformed OABD in executive function, with a small to medium effect size. OABD participants demonstrated on average a 0.53 seconds slower TMT-B performance compared to HC. While the clinical significance of this difference may be context-dependent, it may be enough for a compounding effect when conducting repeated or error-prone tasks that need to be performed under time pressure.

The lack of an age interaction suggests that BD participants who completed the TMT-B test do not experience an accelerated decline in executive function as they age, pointing to other causes for the global cognitive decline observed in OABD, potentially reflecting an endophenotype.

Given that our study provides only a single, cross-sectional snapshot of cognitive performance, it is possible that state-dependent fluctuations (e.g., mood, fatigue, circadian or seasonal variation) or intra-individual variability not captured at one time point contributed to the absence of the expected age interaction. Longitudinal designs may therefore be better suited to detecting whether age-related divergence emerges over time.

Future research should investigate when executive dysfunction first emerges in bipolar disorder, whether early in the course of the disorder or later as individuals age, since our findings are restricted to participants aged 50 years and above. Understanding the timing of these deficits could have important implications for early interventions and remediation strategies. Among the major cognitive remediation strategies are restorative approaches, which aim to improve underlying cognitive processes through structured, repetitive exercises targeting specific executive skills. Compensatory approaches such as for example, the Cognitive Symptom Management and Rehabilitation Therapy (CogSMART) program^37,38^ focus on teaching strategies to work around cognitive deficits, which may involve the use of memory notebooks, calendars, scheduling systems, or digital tools (including AI-based reminders) to support everyday functioning. Functional remediation aims to restore psychosocial functioning by training the use of neurocognitive skills, with an emphasis on practical efficacy in daily life.^39^ Further research is needed to evaluate the effectiveness of these approaches specifically in OABD.

Those participants who were not included in this analysis due to non-completion, might be those more severely affected by neurodegenerative aging-associated processes,^32,34^ despite the fact that our sample was cognitively intact (all but one study excluded dementia). The absence of a group effect when controlling for employment status and psychomotor speed is potentially explainable by overfitting. Further, while employment is an important functional outcome, the variable in our dataset does not differ between voluntary retirement, compulsory retirement, or other reasons for not working. Therefore, lack of employment in OABD should not be interpreted as inability to work or absence of occupational functioning.

### Relationship of Age and Variables Associated With Executive Function Within OABD

As hypothesized, we observed worse executive function associated with older age among OABD participants with small to medium effect sizes. When controlling for psychomotor speed, test-completion was not associated with age, possibly due to a high correlation between psychomotor speed and age.

We expected a relationship between executive function with age of onset, depression and mania severity, antipsychotic and lithium use.^1,9^ Indeed, TMT-B test completion was associated with fewer manic symptoms, but not with other clinical variables, and these results remained unchanged when including TMT-A and/or employment status.

Within test completers, worse executive function was significantly associated with current antipsychotic use, and no other associations in the main model, possibly due to a tendency to prescribe more antipsychotics to BD patients with more severe symptoms.

When covarying for psychomotor speed, additionally, a significant association of worse executive function with more manic symptoms emerged. This suggests that participants with higher manic symptom severity showed more executive dysfunction, specifically in tasks of higher cognitive complexity, such as the TMT-B, rather than in the processing speed or visual scanning components common to both TMT-A and TMT-B.^40^

Our results are in line with literature showing that during a mood episode, individuals exhibited worse cognitive performance compared to when euthymic.^10,41^ However, another study found no association of mania severity with global cognition among OABD participants in manic episodes, who as a group performed more poorly than HC.^42^ These discrepancies might be due to differences in samples as well as cognitive domains assessed.

Previous studies have found lithium beneficial for cognition^39^ In our sample, however, lithium use may reflect factors unrelated to cognition, such as clinical stability.^43^

Although we did not test illness duration directly, in our models that included age of onset we did not see a relationship to executive function.^9^

### Executive Function as a Variable Associated With Daily Functioning and Global Cognition in OABD

As hypothesized, we observed better executive function was associated with better everyday functioning and global cognition. These results were present with or without accounting for antipsychotic use, psychomotor speed or employment status. Executive function could be a treatment target in OABD which, if improved, could result in better function, outcomes, and quality of life for aging BD individuals.

### Strengths and Limitations

The TMT-B primarily assesses cognitive flexibility and completion time depends on cognitive subdomains, such as visual search, visuomotor planning, and processing speed, and thus can still be considered a summary measure. While we controlled for psychomotor speed to reduce confounding, we recognize that more detailed experimental procedures could allow for a finer-grained assessment of specific executive subcomponents. For example, subtasks from the Delis-Kaplan Executive Function System (DKEFS) or computerized paradigms could isolate processes like planning, cognitive flexibility, and response inhibition more precisely. Future studies using such tasks may provide stronger associations and clearer targets to disentangle specific cognitive deficits in older adults with bipolar disorder.

In a cross-sectional study, the result of one test only provides a snapshot. Future studies might want to investigate changes over time and moderators of variability, such as seasonality, hormonal fluctuations or mood state. A further limitation is the use of GAF to assess functioning, which is not specific for older adults.^44^ Additionally, studies involving older adults may be subject to a healthy survivor effect, where individuals who live to older ages tend to be cognitively healthier than the general older population.

Strengths of this project are the large sample size, use of individual harmonized data and the global nature of our dataset that increases generalizability.

## CONCLUSION

Our findings demonstrate that executive function is significantly worse and a key correlate of everyday functioning in OABD. This underscores the clinical need for targeted pro-cognitive interventions—particularly aimed at enhancing and conserving executive abilities—as a critical avenue to improve real-world outcomes. Future studies should aim for a longitudinal design and investigate more diverse executive function assessments and tests that cover additional cognitive domains, such as verbal learning and memory, to cover premorbid cognitive performance, while also including participants with lower cognitive functioning. This will foster the creation of personalized cognitive pharmacological and nonpharmacological treatments for patient subgroups that profit most from targeted treatment interventions.

## Supplementary Material

1

Supplementary material associated with this article can be found in the online version at https://doi.org/10.1016/j.jagp.2025.11.017.

## Figures and Tables

**TABLE 1.** Socio-demographic Characteristics and Comparison Between Older Age Bipolar Disorder and Healthy Comparison Participants

	Total Sample			Healthy Comparisons			Bipolar Disorder			Group Difference Test statistic (Mann-Whitney U or Chi-square test), p-value
	N max 806	M/%	SD/N	N Max192	M/%	SD/N	N max 614	M/%	SD/N	
Age (in years)	806	66.3	8.1	192	65.92	9.58	614	66.5	7.6	*U* = 55,029, p = 0.16
Gender (female)	805	55.20%	444	191	62.30%	119	614	52.9%	325	*χ*^2^=5.17, **p= 0.02**
Education level (years)	763	13.7	3.3	178	13.9	3.2	585	13.7	3.3	*U* = 50,267, p = 0.49
Employment status (Working)	493	27%	133	88	54.50%	48	405	21.00%	85	*χ*^2^=41.4, *p < 0.001*
Executive function(TMT-B completion time)^a^	806	133.3	77.6	192	92.30	49.67	614	146.1	80.26	*U*= 32,472, **p < 0.001**
Executive function test completion ^b^ (TMT-B Completed)	806	89.5%	721	192	99.50%	191	614	86.30%	530	*χ*^2^=26.9, **p < 0.001**
Psychomotor speed (TMT-A completion time) ^c^	682	56.9	39.3	114	43.94	20.28	568	59.51	41.57	*U*= 23,406, **p < 0.001**
Age of disease onset (years)	-	-	-	-	-	-	552	32.8	16.6	-
Depression severity^d^	-	-	-	-	-	-	604			-
No depression								52.20%	315	
Mild to moderate depression								42.90%	259	
Severe depression								5.00%	30	
Manic symptoms (YMRS)	-	-	-	-	-	-	602	10.4	11.7	-
Antipsychotics use (current use)	-	-	-	-	-	-	574	34.10%	196	-
Lithium use (current use)	-	-	-	-	-	-	578	33.70%	195	-
Global Functioning (GAF-score)	-	-	-	-	-	-	391	54	16	-
Global cognition (g-score)^e^	-	-	-	-	-	-	462	0.03	0.99	-

*Notes:* M: mean; SD: standard deviation; BD: Bipolar Disorder; CES-D: Center for Epidemiologic Studies Depression Scale; GAF: Global Assessment of Functioning; HAMD: Hamilton Depression Rating Scale; MDRS: Montgomery-Asberg Depression Rating Scale; YMRS: Young Mania Rating Scale. No covariates are included in descriptive statistics, see text for results of final analyses.

a Trails B raw completion time in seconds, higher numbers indicate worse performance.

b Trails B test completion defined as completion time of 300seconds or less, see text for details.

c Trails A raw completion time in seconds, higher numbers indicate worse performance.

d The depression severity band was harmonized from MDRS, HAMD, and CES-D, see text for cut-offs.

e Cognitive g-score: a continuous z-score scaling metric, see text for details.

**TABLE 2.** Summary of Model Results for TMT-B Performance in OABD versus HC (H1)

Model	Controls Included	OABD vs HC Group Difference	Age × Diagnosis Interaction
M1	Age, education, gender, study (random effect)	+ ↓	–
M2	M1 + TMT-A (psychomotor speed)	+ ↓	–
M3	M1 + Employment Status	+ ↓	–
M4	M2 + Employment Status	–	–

+ = Significant effect (p < 0.05).

− = Not significant (p ≥ 0.05).

→ = Significantly worse executive function in OABD versus HC.

**TABLE 3.** Association Between Age and Executive Function Within OABD Participants (H2a/b)

Model	Covariates Included	Age → Test Completion Status	Age → TMT-B Completion Time
M1	Study, education, gender	+ ↓	+ ↓
M2	M1 + TMT-A (psychomotor speed)	–	+ ↓
M3	M1 + Employment Status	+ ↓	+ ↓
M4	M2 + Employment Status	–	+ ↓

+ = Significant effect (p < 0.05).

− = Not significant (p ≥ 0.05).

↓ = Significant association of younger age with better performance (test completion/ faster TMT-B time).

## Data Availability

Preliminary data from this project was presented at a scientific meeting (ISBD, Japan, September 2025). Data are available as part of the GAGE- BD project and subject to the completion of appropriate data use agreements. Qualified scientists who wish to access the data should contact the study lead author.

## Highlights

- What is the primary question addressed by this study?
  Because executive function deficits are major contributors to disability among older adults with bipolar disorder (OABD), we investigated differences in executive function between OABD and age-equated healthy comparators HC) and examined factors associated with executive function within the OABD group.
- What is the main finding of this study?
  Executive function, assessed by the Trail Making Test B, was worse in OABD than in HC, even after controlling for relevant covariates. Within OABD, executive performance was poorer among those who were older, used antipsychotic medication, had more severe symptoms of mania, and had worse global cognition and daily functioning.
- What is the meaning of the finding?
  Our cross-sectional findings support that executive function is impaired in OABD. Future longitudinal studies of executive function and OABD need to assess the individual impact of impairment in executive function on everyday functioning to inform personalized interventions targeting specific patient subgroups.
