# Supplementary material for: Executive Function Among Older Adults With Bipolar Disorder: A GAGE-BD Analysis
Source: Am J Geriatr Psychiatry. Author manuscript; Available in PMC 2026 Jul 1. (PMC13321887; doi:10.1016/j.jagp.2025.11.017)
Supplement: 1 [file NIHMS2187432-supplement-1.docx]

**Supplemental Data**

**Table S1.
Study site information of contributing studies**

| **Full name of study** | **Sample size (HC/BD)** | **Data Wave** | **Study cohort acronym** | **Site (Country)** | **Study design** |
| --- | --- | --- | --- | --- | --- |
| Atorvastatin for the Treatment of Lithium-Induced Nephrogenic Diabetes Insipidus: A Randomized Controlled Trial | 0/11 | 1 | Atorvastatin | Lady Davis Institute (Canada) | RCT |
| Cognition in Bipolar Disorder XR | 78/119 | 1 | CiBS_XR | GGZ inGeest (Netherlands) | Observational |
| Cognition in Euthymic Older Adults with Bipolar Disorder | 0/47 | 1 | Cog-BD | Center for Addiction & Mental Health (Canada) | Observational |
| Dutch Older Bipolar cohort wave 1 | 0/48 | 1 | DOBi1 | GGZ inGeest (Netherlands) | Observational |
| Dutch Older Bipolar cohort wave 2 | 0/61 | 1 | DOBi2 | GGZ inGeest (Netherlands) | Observational |
| Acute Pharmacotherapy of Late-Life Mania | 0/193 | 2 | GERI-BD | Multi-site/Case Western Reserve University (USA) | RCT |
| Open-label, Prospective Trial of Lamotrigine for Symptoms of Geriatric Bipolar Depression | 0/23 | 1 | GERI-SAD | Case Western Reserve University (USA) | Prospective uncontrolled intervention with Lamotrigine |
| Dynamic Inflammatory and Mood Predictors of Cognitive Aging in Bipolar Disorder | 32/29 | 1 | Inflammaging | University of California San Diego (USA) | Observational |
| Geriatric Psychiatry Mood Disorders Research Database | 23/35 | 1 | GMDD | McLean Hospital (USA) | Observational |
| Asenapine in the Treatment of Older Adults With Bipolar Disorder | 0/14 | 1 | OPT-BD | Case Western Reserve University (USA) | Prospective uncontrolled intervention with Asenapine |
| The Effect of Bipolar Disorder and its Comorbidities on Cognition in Older Adults | 13/19 | 1 | UPMC | University of Pittsburgh Medical Center (USA) | Observational |
| Structural and Functional Brain Aging in Bipolar Disorder | 46/15 | 2 | VA_BAI | Veterans Affairs San Diego Healthcare System (USA) | Observational |

**Notes:**RCT: Randomized controlled trial. For the current analysis, only participants with OABD (≥50 years) and available data from the Trails B test were included.

**Table S2.**
**Inclusion and exclusion criteria for each contributing study site**

| **Wave 1 Study cohort acronym** | **Inclusion criteria** | **Exclusion criteria** |
| --- | --- | --- |
| Atorvastatin | 1. Individuals of 18 years of age or older (including patients 18-64 and 65+, with no maximum age limit)  2. Individuals with bipolar disorder in any phase of illness: euthymic, depressed, or hypomanic. Patients were recruited from the outpatient bipolar and geriatric psychiatry clinics  3. Able and willing to give informed consent  4. Chronic and current lithium users (at least 2 months of Lithium use)  5. Stable dose of lithium for the past 2 months. - Patients taking any lithium level will be included  6. In the original study, patients with any psychiatric diagnosis were included, and had either bipolar disorder (n=54) or unipolar depression (n=6). Only patients with bipolar disorder who re-consented to data sharing with GAGE-BD were included in the current GAGE-BD analysis.  7. Patients were included in the atorvastatin trial if they had partial or complete nephrogenic diabetes insipidus (NDI) - defined as a 10-hour water restriction urine osmolality (UOsm) ≤300mOsm/Kg | 1. Patients with statin use within 6 weeks prior to the study  2. Patients with a history of severe adverse reaction to statins  3. Patients with a baseline Low Density Lipoprotein (LDL) level <1.5  4. Relative contraindications to statin use 42: pregnancy or lactation, concurrent use of fibrates, heavy ethanol consumption (>50 units/week)  5. Incapacity to consent  6. Deemed by the treating physician to have a severe cognitive or behavioural disturbance such as acute delirium or moderate-severe DSM5 Neurocognitive Disorder (dementia), preventing their ability to complete safely the study questionnaire and/or to provide blood and urine test. |
| CiBS_XR | 1. 60+ years old  2. Diagnosis of bipolar I or bipolar II  3. Outpatients  4. Euthymic for at least 3 weeks as assessed by patient's psychiatrist  5. No history of ECT  *Comparison group:*  1. 60+ years old  2. No current or lifetime psychiatric illness or addict | 1. Not euthymic as assessed by YMRS, CESD  2. History of ECT  3. Alcohol dependency or substance abuse  4. Dementia  *Comparison group:*  1. History of psychiatric illness or addiction  2. Recent memory complaint |
| Cog-BD | 1. Age 50 years and above  2. Meets DSM-IV TR criteria for a current diagnosis of Bipolar I or II Disorder  3. Willingness and ability to speak English  4. Willingness to provide informed consent  5. Corrected visual ability that enables reading of newspaper headlines and hearing capacity that is adequate to respond to a raised conversational voice.  6. At time of assessment they should be clinically euthymic for four weeks preceding study entry, with both HRSD-17 and YMRS scores of 10 or less at time of assessment (The criteria are selected to capture bipolar disorder across the older adults. We are interested in bipolar I and II disorder to capture bipolar illness that would generalize to "real-world," clinical practice. Although there are no set criteria for designating "acceptable" euthymia by HRSD-17 and YMRS, there is growing consensus among geriatric psychiatrists that scores 10 or less on these instruments are indicated to minimize acute impact of mood symptoms when performing NP testing). | 1. Does not meet criteria for any type of dementia or other neurological disorder affecting the central nervous system (for example, multiple sclerosis, history of traumatic brain injury, cerebrovascular disease)  2. No history of schizophrenia, schizoaffective or other psychotic disorders  3. No alcohol or other drug abuse/dependence within 6 months of testing  4. No Electroconvulsive Therapy (ECT) within 6 months of testing. |
| DOBi1/2 | 1. Age ≥ 60 years (DOBi1), ≥ 50 years (DOBi2) 2. Diagnostic procedure (MINI) indicates BD-I, BD-II, or BD-NOS  3. Willing to give consent (some consented to chart review plus structured interview, others consented to chart review only) | 1. Dementia  2. Intellectual disability (IQ<70)  3. Language barrier  4. Poor cognition (measured by Mini Mental State Examination; MMSE <18)  5. Insufficiently stable psychiatric condition |
| GERI-SAD | 1. Age > 60 Years  2. BP Disorder-I or II: Depressive episode (DSM -IV-TR; SCID-I/P)  3. HAM-D > 18 (GRIDHAM-D 24-item version)  4. Availability of an informant is encouraged but not required for study participation. | 1. Chronic psychotic conditions, ie. schizophrenia, schizoaffective disorder, delusional disorder 2. Contraindication to lamotrigine (Physician interview, medical assessment)  3. Documented history of intolerance to lamotrigine  4. Patients who have previously failed to respond to at least 12 weeks of treatment with lamotrigine  5. Active substance dependence (SCID-I/P) or substance-related safety issues or PI concerns  6. Mood Disorder Due to a General Medical Condition or Treatment (Physician interview)  7. Rapid cycling (Physician interview): As defined in DSM-IV: At least 4 episodes of mood disturbance in the previous 12 months that meet criteria for a Major Depressive, Manic, Mixed or Hypomanic Episode. Episodes are distinguished either by partial or full remission for at least 2 months or by a switch to an episode of opposite polarity  8. Dementia (by DSM-IV or brain degenerative diseases; Physician interview)  9. Inability to communicate in English (i.e., interview cannot be conducted without an interpreter; subject  largely unable to understand questions and cannot respond in English)  10. Clinically significant sensory impairment (i.e., cannot see well enough to read consent or visually presented material; cannot hear well enough to cooperate with interview; Physician interview)  11. Recent history of cardiovascular, peripheral vascular events or stroke 12. High risk for suicide (e.g., active SI or current intent or plan)  13. Inpatient status. |
| Inflammaging | 1. Diagnosis of Bipolar I or II Disorder by DSM-IV criteria  2. Age 25-60 years, currently outpatient, proficient in English 3. Capable of providing informed consent. | 1. Acute medical illness (e.g., cold, flu, bacterial infection, heart failure, cancer) or pregnancy  2. Recent (<6 weeks) vaccination  3. History of neurological disorder (e.g., dementia, seizures, Parkinson's, stroke) or head trauma with unconsciousness > 15 minutes  4. Diagnosis of substance abuse within the last 3 months or dependence within the last 6 months  5. History of radiation or chemotherapy treatment,  uncontrolled diabetes or hypertension, sensory limitations including vision uncorrectable to 20/40, conservatorized, color blindness or hearing loss that interferes with assessment, chronic pain that necessitates treatment with nonsteroidal anti-inflammatory drugs or prescription painkillers that would affect blood-based markers of inflammation. |
| GMDD | 1. Diagnosis of Major Depressive Disorder, Depression NOS, Dysthymia, Bipolar Disorder, Type I or II, Bipolar Depression NOS, or any other diagnosis of a mood disorder  2. Ability to provide Informed Consent  3. Age 55-89, inclusive | 1. Inability to complete diagnostic assessment (not including intelligence testing)  2. Inability to speak English  3. Active substance abuse or dependence (other than caffeine or nicotine) within the last month as determined by SCID DSM-IV, MINI, or clinical judgment. |
| OPT-BD | 1. Subjects must have type I Bipolar disorder by DSM-IV  criteria confirmed on the Mini Neuropsychiatric Interview (MINI)  2. Subjects must be age 60 or older  3. Subjects must have suboptimal response to current psychotropic management including at least one of the following:  a. Behaviors and symptoms of irritability, agitation, mood lability or diminished ability to interact with others in their place of residence b. Diminished ability to take care of basic personal needs in their place of residence due to symptoms of BD | 1. History of intolerance or resistance to Asenapine  2. Clinical diagnosis of dementia or Mini-mental state (MMSE) < 24  3. History of TIA, stroke or MI within the past 12 months  4. Medical illness that is the clear, underlying etiology of BD  5. Unstable medical illness or condition including prolonged QT interval, which in the opinion of the study investigators, is likely to affect the outcome of the study or the subject's safety  6. DSM-IV substance dependence (except nicotine or caffeine) within the past 3 months.  7. Rapid cycling BD defined as 4 or more discrete mood episodes within the previous 12 months.  8. At high risk for self-harm or suicide |
| UPMC | 1. Age ≥ 50 years 2. Clinical euthymia for four weeks preceding neurocognitive assessment with scores of ≤ 10 on both the 17-item Hamilton  Rating Scale for Depression (HRSD) and the Young Mania  Rating Scale (YMRS) at the time of assessment  3. Ability to comprehend and speak English fluently  4. Corrected visual ability to read newspaper headlines  5. Hearing capacity adequate to respond to a raised  conversational voice | 1. History of dementia or neurologic disorder affecting the central nervous system (e.g., Parkinson's  disease, traumatic brain injury, or multiple sclerosis)  2. Electroconvulsive therapy within the past six months  3. Substance abuse or dependence within the past 12 months.  4. For this report, we focused on subjects who had completed both neuroimaging and neurocognitive assessment. |
| **Wave 2 Study cohort acronym** | **Inclusion criteria** | **Exclusion criteria** |
| GERI-BD | 1. Age greater than or equal to 60 years  2. Bipolar Disorder, Type I: Current Manic, Mixed or Hypomanic Episode as defined by the Diagnostic and Statistical Manual for Mental Disorders - Fourth Edition and as confirmed by Structured Clinical Interview for DSM-IV Disorders (SCID-P).  3. Young Mania Rating Scale (YMRS) total score greater than or equal to 18. | 1. Chronic psychotic conditions (i.e., schizophrenia, schizoaffective disorder).  2. Contraindication to study medications  3. Documented history of intolerance of LI, DV, lorazepam, or risperdone.  4. Patients who have failed to respond to at least 4 weeks of treatment with LI (greater than or equal to 0.4mEq/L) or VAL (greater than or equal to 40 ug/ml)  5. Active substance dependence or any substance-related safety issues or PI concerns.  6. Mood Disorder Due to a General Medical Condition or Treatment. Patients with mania associated with use of steroids, L-DOPA use, recent stroke, hyperthyroidism, porphyria, HIV infection, and connective tissue diseases, are excluded because the proposed interventions may be insufficient for their treatment.  7. Patients with rapid cycling BP disorder will be excluded, because these patients may require different treatments and should be studied separately.  8. Dementia and brain degenerative diseases.  9. Delirium;  10. Inability to communicate in English. Communication in languages other than English, because many of the primary assessment tools have not been validated in other languages.  11. Clinically significant sensory impairment. Auditory acuity, and visual acuity that allows for reading words, are necessary for testing global cognitive performance.  12. Recent history of cardiovascular, peripheral vascular events, or stroke.  13. High risk for suicide (e.g., active suicidal ideation and current intent or plan) in an ambulatory patient. |
| VA_BAI | 1. Ages 30-79 years old.  *Patients:*  2. DSM-IV diagnosis of BD and onset of first mood episode between ages 13 to 35 years. | *Patients:*  1. Current depressive or manic episode as determined by DSM-IV criteria or significant residual mood or psychotic symptoms, change of medication or dose in past 6 weeks, other co-morbid Axis I disorder (anxiety disorder allowed if no symptoms or treatment within 1 year).  2. Current or recent (past 6 months for abuse, past 12 months for dependence) diagnosis of substance abuse or dependence,  3. History of head injury with loss of consciousness for > 30 minutes, left handedness, history of neurological disorder (e.g., seizure disorder, Parkinson’s or Alzheimer’s disease, stroke).  4. History of diabetes, uncontrolled hypertension  5. Contraindications for MRI scanning (e.g., metal in the body, weight over 300 lbs, claustrophobia, difficulty lying still, pregnancy).  6. Native language other than English.  7. Conservatorized.  8. Children under the age of 18 will be excluded due to the aims of the study to examine adult age-related changes.  *Healthy individuals:*  1. Current Axis I disorder as determined by the Mini-International Neuropsychiatric Interview (MINI 4.0), first-degree relatives with bipolar disorder, unipolar depression, or schizophrenia.  2. All the non-psychiatric exclusion criteria listed above. |
|  |  |  |

|  | **Total Sample Bipolar Disorder** | | | **Executive function test non-completers** | | | **Executive function test completers** | | | **Group Difference** |
| --- | --- | --- | --- | --- | --- | --- | --- | --- | --- | --- |
|  | **N**  max 614 | **M/%** | **SD/N** | **N**  Max  84 | **M/%** | **SD/N** | **N**  max 530 | **M/%** | **SD/N** | **Test statistic (Mann-Whitney U or**  **Chi-square test), p-value** |
| Age (in years) | 614 | 66.5 | 7.6 | 84 | 70.9 | 7.7 | 530 | 65.8 | 7.4 | U=13959, **p<0.001** |
| Gender (Female) | 614 | 52.9% | 325 | 84 | 57.1% | 48 | 530 | 52.3% | 277 | χ^2^=693, p=.41 |
| Education level (years) | 585 | 13.7 | 3.3 | 79 | 11.6 | 3.6 | 506 | 14.0 | 3.2 | U=11677, **p<0.001** |
| Employment status (working) | 405 | 21.0% | 85 | 52 | 7.7% | 4 | 353 | 22.9% | 81 | χ^2^=6.4, **p=.01** |
| Executive function (TMT-B completion time) ^a^ | 614 | 146.1 | 80.26 | 84 | 300.0 | 0.0 | 530 | 121.7 | 55.7 | U=0, **p<0.001** |
| Psychomotor speed (TMT- A completion time) ^c^ | 568 | 59.51 | 41.57 | 81 | 119.0 | 70.3 | 487 | 49.6 | 22.7 | U=4931,  **p<0.001** |
| Age of disease onset (years) | 552 | 32.8 | 16.6 | 77 | 38.2 | 19.6 | 475 | 32.0 | 15.9 | - ^f^ |
| Depression severity ^d^  No depression  Mild to moderate depression  Severe depression | 604 | 52.2%  42.9%  5.0% | 315  259  30 | 84 | 50.0%  46.4%  3.6% | 42  39  3 | 520 | 52.5%  42.3%  5.2% | 273  220  27 | - ^f^ |
| Manic symptoms (YMRS) | 602 | 10.4 | 11.7 | 83 | 14.5 | 14.2 | 519 | 9.8 | 11.1 | - ^f^ |
| Antipsychotics use (current use) | 574 | 34.1% | 196 | 78 | 35.9% | 28 | 496 | 33.9% | 168 | - ^f^ |
| Lithium use (current use) | 578 | 33.7% | 195 | 78 | 34.6% | 27 | 500 | 33.6% | 168 | - ^f^ |
| Global Functioning (GAF-score) | 391 | 54 | 16 | 50 | 45.0 | 16.0 | 341 | 55.0 | 16.0 | - ^f^ |
| Global cognition (g-score) ^e^ | 462 | 0.03 | 0.99 | 47 | -1.5 | 0.9 | 415 | 0.1 | 0.9 | - ^f^ |

**S3: Sample characteristics within older age bipolar group**

**Notes:** M = mean; SD = standard deviation; BD = Bipolar Disorder; CES-D = Center for Epidemiologic Studies Depression Scale; GAF = Global Assessment of Functioning; HAMD = Hamilton Depression Rating Scale; MDRS = Montgomery-Asberg Depression Rating Scale; YMRS = Young Mania Rating Scale.
^a^ Trails B raw completion time in seconds, higher numbers indicate worse performance

^b^ Trails B test completion defined as completion time of 300seconds or less, see text for details

^c^ Trails A raw completion time in seconds, higher numbers indicate worse performance

^d^ The depression severity band was harmonized from MDRS, HAMD, and CES-D, see text for cut-offs.
^e^ Cognitive g-score: a continuous z-score scaling metric, see text for details.

^f^ Results of these comparisons will be presented in the text, see respective results section.

**S4: Summary of Models for Associations Between Clinical Variables and Executive Function Within OABD TMT-B Test Completers (H2c)**

| **Model** | **Covariates Included** | **Depression Severity** | **Age of Onset** | **Lithium Use** | **Mania Severity** | **Antipsychotic Use** |
| --- | --- | --- | --- | --- | --- | --- |
| M1 | Age, education, gender, study (random effect) | – | – | – | – | +↓ |
| M2 | M1 + TMT-A | – | – | – | + ↓ | +↓ |
| M3 | M1 + Employment Status | – | – | – | - | +↓ |
| M4 | M2 + Employment Status | – | – | – | – | – |

+ = indicates significant (FDR-adjusted p < .05); "–" indicates non-significant (p ≥ .05).

↓ = Significant association with **better executive function** (e.g., no antipsychotic use/lower mania severity)

**S5: Summary of Models for Associations Between Clinical Variables and TMT-B Test Completion in OABD Participants (H2d)**

| **Model** | **Covariates Included** | **Mania Severity** | **Age of Onset** | **Depression Severity** | **Lithium Use** | **Antipsychotic Use** |
| --- | --- | --- | --- | --- | --- | --- |
| M1 | Age, education, gender, study (random effect) | +↓ | – | – | – | – |
| M2 | M1 + TMT-A | +↓ | – | – | – | – |
| M3 | M1 + Employment Status | +↓ | – | – | – | – |
| M4 | M2 + Employment Status | +¯ | – | – | – | – |

+ indicates significant (FDR-adjusted p < .05)

– indicates non-significant (p ≥ .05)

↓ = Significant association with **better executive function** (lower mania severity)

**S6: Summary of Models for Associations of Executive Function with Functioning and Global Cognition in OABD TMT-B Test Completers (H2e/f)**

| **Model** | **Covariates Included** | **Functioning** | **Global Cognition** |
| --- | --- | --- | --- |
| M1a | Study, age, education, gender + antipsychotic use | + ­↑ | + ­↑ |
| M1b | Study, age, education, gender (no antipsychotics) | + ­↑ | + ­↑ |
| M2 | M1 + TMT-A | + ­↑ | + ­↑ |
| M3 | M1 + Employment Status | + ­↑ | + ­↑ |
| M4 | M2 + Employment Status | + ­↑ | + ­↑ |

+ = Significant effect (p < 0.05)

– = Not significant (p ≥ 0.05)

↑ ­= Significant association with **better executive function** (lower mania severity)

**S7: Coefficients of models**

**H1-M1: Coefficients for TMT-B Performance in OABD vs HC (without age* group interaction)**

| **Parameter** | **Estimate** | **Std. Error** | **df** | **t** | **Sig.** | **95% Confidence Interval** | |  |
| --- | --- | --- | --- | --- | --- | --- | --- | --- |
|  |  |  |  |  |  | **Lower Bound** | **Upper Bound** | **Partial Eta Squared ^1^** |
| Intercept | -1.73 | 0.34 | 429.36 | -5.15 | <.001 | -2.39 | -1.07 | 0.13 |
| age | 0.04 | 0.00 | 224.32 | 10.20 | <.001 | 0.04 | 0.05 | 0.13 |
| education | -0.07 | 0.01 | 289.98 | -6.21 | <.001 | -0.09 | -0.05 | 0.07 |
| Gender [men] | -0.11 | 0.07 | 676.45 | -1.64 | 0.10 | -0.24 | 0.02 | 0.00 |
| Group [HC] | -0.57 | 0.08 | 105.75 | -7.04 | <.001 | -0.73 | -0.41 | 0.05 |

Estimates of fixed effects derived from linear mixed models, random effect: study

Gender reference is women, diagnostic group reference is BD.

^1^ Partial eta squared derived from GLM including study as covariate.

**H1-M1: Coefficients for TMT-B Performance in OABD vs HC (with age* group interaction)**

| **Parameter** | **Estimate** | **Std. Error** | **df** | **t** | **Sig.** | **95% Confidence Interval** | |  |  |  |
| --- | --- | --- | --- | --- | --- | --- | --- | --- | --- | --- |
|  |  |  |  |  |  | **Lower Bound** | **Upper Bound** | **Partial Eta Squared ^1^** |  |  |
| Intercept | -1.94 | 0.38 | 558.01 | -5.07 | <.001 | -2.69 | -1.19 | 0.11 |  |  |
| age | 0.05 | 0.01 | 484.63 | 9.08 | <.001 | 0.04 | 0.06 | 0.11 |  |  |
| education | -0.07 | 0.01 | 281.10 | -6.27 | <.001 | -0.09 | -0.05 | 0.07 |  |  |
| Gender [men] | -0.11 | 0.07 | 674.60 | -1.71 | 0.09 | -0.24 | 0.02 | 0.001 |  |  |
| Group [HC] | 0.07 | 0.56 | 624.95 | 0.13 | 0.90 | -1.03 | 1.17 | 0.001 |  |  |
| Group [HC] * age | -0.01 | 0.01 | 650.22 | -1.15 | 0.25 | -0.03 | 0.01 | 0.001 |  |  |
|  |  |  |  |  |  |  |  |  |  |  |

Estimates of fixed effects derived from linear mixed models, random effect: study

Gender reference is women, diagnostic group reference is BD.

^1^ Partial eta squared derived from GLM including study as covariate.

**H1-M2: Coefficients for TMT-B Performance in OABD vs HC**

| Parameter | Estimate | Std. Error | df | t | Sig. | 95% Confidence Interval | |  |
| --- | --- | --- | --- | --- | --- | --- | --- | --- |
|  |  |  |  |  |  | Lower Bound | Upper Bound | **Partial Eta Squared ^1^** |
| Intercept | -0.77 | 0.33 | 505.54 | -2.35 | 0.02 | -1.41 | -0.13 | 0.14 |
| age | 0.02 | 0.00 | 548.98 | 5.27 | <.001 | 0.01 | 0.03 | 0.06 |
| education | -0.04 | 0.01 | 476.75 | -3.93 | <.001 | -0.06 | -0.02 | 0.08 |
| Psychomotor speed (TMT-A) | 0.50 | 0.03 | 556.32 | 15.03 | <.001 | 0.43 | 0.56 | 0.27 |
| Gender [men] | -0.16 | 0.06 | 556.92 | -2.67 | 0.01 | -0.28 | -0.04 | 0.01 |
| Group [HC] | -0.33 | 0.09 | 353.56 | -3.61 | <.001 | -0.52 | -0.15 | 0.02 |

Estimates of fixed effects derived from linear mixed models, random effect: study

Gender reference is women, diagnostic group reference is BD.

^1^ Partial eta squared derived from GLM including study as covariate.

**H1-M3: Coefficients for TMT-B Performance in OABD vs HC**

| **Parameter** | **Estimate** | **Std. Error** | **df** | **t** | **Sig.** | **95% Confidence Interval** | |  |
| --- | --- | --- | --- | --- | --- | --- | --- | --- |
|  |  |  |  |  |  | **Lower Bound** | **Upper Bound** | **Partial Eta Squared ^1^** |
| Intercept | -1.80 | 0.43 | 265.59 | -4.20 | <.001 | -2.64 | -0.95 | 0.04 |
| age | 0.05 | 0.01 | 284.52 | 7.70 | <.001 | 0.03 | 0.06 | 0.11 |
| education | -0.09 | 0.01 | 381.99 | -6.44 | <.001 | -0.12 | -0.06 | 0.07 |
| Gender [men] | -0.12 | 0.08 | 427.97 | -1.48 | 0.14 | -0.28 | 0.04 | . 005 |
| Occupation status [not employed] | 0.25 | 0.10 | 428.30 | 2.65 | 0.01 | 0.06 | 0.44 | 0.02 |
| Group [HC] | -0.34 | 0.12 | 55.70 | -2.80 | 0.01 | -0.58 | -0.10 | 0.01 |

Estimates of fixed effects derived from linear mixed models, random effect: study

Gender reference is women, diagnostic group reference is BD, occupation reference is being employed.

^1^ Partial eta squared derived from GLM including study as covariate.

**H1-M4: Coefficients for TMT-B Performance in OABD vs HC**

| **Parameter** | **Estimate** | **Std. Error** | **df** | **t** | **Sig.** | **95% Confidence Interval** | |  |
| --- | --- | --- | --- | --- | --- | --- | --- | --- |
|  |  |  |  |  |  | **Lower Bound** | **Upper Bound** | **Partial Eta Squared ^1^** |
| Intercept | -0.91 | 0.44 | 278.52 | -2.08 | 0.04 | -1.77 | -0.05 | 0.06 |
| age | 0.03 | 0.01 | 325.98 | 4.22 | <.001 | 0.01 | 0.04 | 0.05 |
| education | -0.06 | 0.01 | 315.35 | -4.28 | <.001 | -0.08 | -0.03 | 0.05 |
| Psychomotor speed (TMT-A) | 0.45 | 0.04 | 324.89 | 10.88 | <.001 | 0.37 | 0.53 | 0.25 |
| Gender [men] | -0.22 | 0.08 | 324.73 | -2.90 | 0.00 | -0.38 | -0.07 | 0.03 |
| Occupation status [not employed] | 0.29 | 0.09 | 325.32 | 3.08 | 0.00 | 0.10 | 0.47 |  |
| Group [HC] | -0.91 | 0.44 | 278.52 | -2.08 | 0.04 | -1.77 | -0.05 | 0.06 |

Estimates of fixed effects derived from linear mixed models, random effect: study

Gender reference is women, diagnostic group reference is BD, occupation reference is being employed.

^1^ Partial eta squared derived from GLM including study as covariate.

**H2a-M1: Association Between Age and Executive Function (Test completion status) Within OABD Participants**

| Parameter | Estimate | Std. Error | df | t | Sig. | 95% Confidence Interval | |  |
| --- | --- | --- | --- | --- | --- | --- | --- | --- |
|  |  |  |  |  |  | Lower Bound | Upper Bound | **Partial Eta Squared ^1^** |
| Intercept | 65.23 | 1.88 | 38.81 | 34.70 | <.001 | 61.43 | 69.03 | 0.07 |
| education | -0.05 | 0.09 | 578.95 | -0.54 | 0.59 | -0.24 | 0.13 | . 045 |
| Gender [men] | -0.68 | 0.57 | 575.35 | -1.19 | 0.24 | -1.81 | 0.45 | 0.00 |
| Executive functioning (TMT-B test non-completer) | 2.80 | 0.86 | 573.11 | 3.27 | 0.00 | 1.12 | 4.48 | 0.02 |

Estimates of fixed effects derived from linear mixed models, random effect: study

Gender reference is women, test completion reference is completion of TMT-B.

^1^ Partial eta squared derived from GLM including study as covariate.

**H2a-M2: Association Between Age and Executive Function (Test completion status) Within OABD Participants**

| Parameter | Estimate | Std. Error | df | t | Sig. | 95% Confidence Interval | |  |
| --- | --- | --- | --- | --- | --- | --- | --- | --- |
|  |  |  |  |  |  | Lower Bound | Upper Bound | **Partial Eta Squared ^1^** |
| Intercept | 63.18 | 1.74 | 68.84 | 36.39 | <.001 | 59.72 | 66.64 | 0.08 |
| education | 0.00 | 0.10 | 534.04 | 0.04 | 0.97 | -0.18 | 0.19 | 0.00 |
| Gender [men] | -0.39 | 0.58 | 531.70 | -0.67 | 0.51 | -1.54 | 0.76 | 0.00 |
| Psychomotor speed (TMT A) | 0.05 | 0.01 | 528.30 | 6.41 | <.001 | 0.04 | 0.07 | 0.28 |
| Executive functioning (TMT-B test non-completer) | -0.51 | 1.02 | 530.38 | -0.50 | 0.62 | -2.52 | 1.50 | 0.00 |

Estimates of fixed effects derived from linear mixed models, random effect: study

Gender reference is women, test completion reference is completion of TMT-B.

^1^ Partial eta squared derived from GLM including study as covariate.

**H2a-M3: Association Between Age and Executive Function (Test completion status) Within OABD Participants**

| Parameter | Estimate | Std. Error | df | t | Sig. | 95% Confidence Interval | |  |
| --- | --- | --- | --- | --- | --- | --- | --- | --- |
|  |  |  |  |  |  | Lower Bound | Upper Bound | **Partial Eta Squared ^1^** |
| Intercept | 60.36 | 2.27 | 36.23 | 26.56 | <.001 | 55.75 | 64.97 | 0.08 |
| education | 0.13 | 0.11 | 392.27 | 1.24 | 0.22 | -0.08 | 0.34 | 0.04 |
| Gender [men] | -0.65 | 0.64 | 389.94 | -1.02 | 0.31 | -1.92 | 0.61 | 0.00 |
| Occupation status [not employed] | 2.87 | 0.78 | 387.87 | 3.67 | <.001 | 1.33 | 4.41 | 0.00 |
| Executive functioning (TMT-B test non-completer) | 2.49 | 0.96 | 387.99 | 2.59 | 0.01 | 0.60 | 4.38 | 0.02 |

Estimates of fixed effects derived from linear mixed models, random effect: study

Gender reference is women, test completion reference is completion of TMT-B, occupation reference is being employed.

^1^ Partial eta squared derived from GLM including study as covariate.

**H2a-M4: Association Between Age and Executive Function (Test completion status) Within OABD Participants**

| Parameter | Estimate | Std. Error | df | t | Sig. | 95% Confidence Interval | |  |
| --- | --- | --- | --- | --- | --- | --- | --- | --- |
|  |  |  |  |  |  | Lower Bound | Upper Bound | **Partial Eta Squared ^1^** |
| Intercept | 59.24 | 1.96 | 140.50 | 30.28 | <.001 | 55.37 | 63.11 | 0.09 |
| education | 0.19 | 0.11 | 338.05 | 1.74 | 0.08 | -0.02 | 0.40 | 0.01 |
| Gender [men] | -0.55 | 0.66 | 353.86 | -0.82 | 0.41 | -1.85 | 0.76 | 0.00 |
| Psychomotor speed (TMT A) | 0.05 | 0.01 | 352.79 | 4.40 | <.001 | 0.03 | 0.07 | 0.30 |
| Occupation status [not employed] | 2.94 | 0.83 | 354.79 | 3.56 | <.001 | 1.32 | 4.56 | 0.00 |
| Executive functioning (TMT-B test non-completer) | -0.03 | 1.19 | 354.80 | -0.02 | 0.98 | -2.36 | 2.31 | 0.00 |

Estimates of fixed effects derived from linear mixed models, random effect: study

Gender reference is women, test completion reference is completion of TMT-B, occupation reference is being employed.

^1^ Partial eta squared derived from GLM including study as covariate.

**H2b-M1: Association Between Age and Executive Function (Test completion time) Within OABD Participants**

| Parameter | Estimate | Std. Error | df | t | Sig. | 95% Confidence Interval | |  |
| --- | --- | --- | --- | --- | --- | --- | --- | --- |
|  |  |  |  |  |  | Lower Bound | Upper Bound | **Partial Eta Squared ^1^** |
| Intercept | -1.88 | 0.40 | 393.89 | -4.67 | <.001 | -2.68 | -1.09 | 0.05 |
| age | 0.05 | 0.01 | 294.94 | 8.74 | <.001 | 0.04 | 0.06 | 0.11 |
| education | -0.07 | 0.01 | 333.01 | -5.43 | <.001 | -0.09 | -0.04 | 0.04 |
| Gender [men] | -0.19 | 0.08 | 497.01 | -2.44 | 0.02 | -0.34 | -0.04 | 0.01 |

Estimates of fixed effects derived from linear mixed models, random effect: study

Gender reference is women, diagnostic group reference is BD.

^1^ Partial eta squared derived from GLM including study as covariate.

**H2b-M2: Association Between Age and Executive Function (Test completion time) Within OABD Participants**

| Parameter | Estimate | Std. Error | df | t | Sig. | 95% Confidence Interval | |  |
| --- | --- | --- | --- | --- | --- | --- | --- | --- |
|  |  |  |  |  |  | Lower Bound | Upper Bound | **Partial Eta Squared ^1^** |
| Intercept | -0.90 | 0.37 | 420.17 | -2.42 | 0.02 | -1.64 | -0.17 | 0.06 |
| age | 0.02 | 0.01 | 449.18 | 4.79 | <.001 | 0.01 | 0.03 | 0.05 |
| education | -0.04 | 0.01 | 424.96 | -3.17 | 0.00 | -0.06 | -0.01 | 0.02 |
| Psychomotor speed (TMT-A) | 0.48 | 0.04 | 458.69 | 12.91 | <.001 | 0.40 | 0.55 | 0.22 |
| Gender [men] | -0.20 | 0.07 | 458.92 | -2.91 | 0.00 | -0.33 | -0.06 | 0.02 |

Estimates of fixed effects derived from linear mixed models, random effect: study

Gender reference is women, diagnostic group reference is BD.

^1^ Partial eta squared derived from GLM including study as covariate.

**H2b-M3: Association Between Age and Executive Function (Test completion time) Within OABD Participants**

| Parameter | Estimate | Std. Error | df | t | Sig. | 95% Confidence Interval | |  |
| --- | --- | --- | --- | --- | --- | --- | --- | --- |
|  |  |  |  |  |  | Lower Bound | Upper Bound | **Partial Eta Squared ^1^** |
| Intercept | -2.01 | 0.50 | 244.18 | -4.03 | <.001 | -2.99 | -1.02 | 0.04 |
| age | 0.05 | 0.01 | 256.06 | 6.91 | <.001 | 0.03 | 0.06 | 0.11 |
| education | -0.09 | 0.02 | 298.83 | -5.84 | <.001 | -0.12 | -0.06 | 0.07 |
| Gender [men] | -0.19 | 0.09 | 341.04 | -2.04 | 0.04 | -0.37 | -0.01 | 0.01 |
| Occupation status [not employed] | 0.34 | 0.11 | 341.42 | 3.08 | 0.00 | 0.12 | 0.55 | 0.03 |

Estimates of fixed effects derived from linear mixed models, random effect: study

Gender reference is women, diagnostic group reference is BD, occupation reference is being employed.

^1^ Partial eta squared derived from GLM including study as covariate.

**H2b-M4: Association Between Age and Executive Function (Test completion time) Within OABD Participants**

| Parameter | Estimate | Std. Error | df | t | Sig. | 95% Confidence Interval | |  |
| --- | --- | --- | --- | --- | --- | --- | --- | --- |
|  |  |  |  |  |  | Lower Bound | Upper Bound | **Partial Eta Squared ^1^** |
| Intercept | -0.96 | 0.47 | 264.82 | -2.04 | 0.04 | -1.88 | -0.03 | 0.06 |
| age | 0.03 | 0.01 | 305.99 | 4.01 | <.001 | 0.01 | 0.04 | 0.05 |
| education | -0.06 | 0.01 | 296.08 | -4.09 | <.001 | -0.08 | -0.03 | 0.05 |
| Psychomotor speed (TMT-A) | 0.44 | 0.04 | 304.99 | 10.30 | <.001 | 0.36 | 0.53 | 0.23 |
| Gender [men] | -0.21 | 0.08 | 304.91 | -2.66 | 0.01 | -0.37 | -0.06 | 0.02 |
| Occupation status [not employed] | -0.96 | 0.47 | 264.82 | -2.04 | 0.04 | -1.88 | -0.03 | 0.06 |

Estimates of fixed effects derived from linear mixed models, random effect: study

Gender reference is women, diagnostic group reference is BD, occupation reference is being employed.

^1^ Partial eta squared derived from GLM including study as covariate.

**H2c-M1: Associations Between Clinical Variables and Executive Function Within OABD TMT-B Test Completers**

**Mania Severity**

| Parameter | Estimate | Std. Error | df | t | Sig. | 95% Confidence Interval | |  |
| --- | --- | --- | --- | --- | --- | --- | --- | --- |
|  |  |  |  |  |  | Lower Bound | Upper Bound | **Partial Eta Squared ^1^** |
| Intercept | -1.99 | 0.40 | 310.54 | -4.93 | <.001 | -2.79 | -1.20 | 0.06 |
| age | 0.05 | 0.01 | 209.89 | 8.76 | <.001 | 0.04 | 0.06 | 0.12 |
| education | -0.07 | 0.01 | 239.15 | -5.44 | <.001 | -0.09 | -0.04 | 0.04 |
| Gender [men] | -0.20 | 0.08 | 482.92 | -2.60 | 0.01 | -0.36 | -0.05 | 0.01 |
| Mania severity (YMRS) | 0.01 | 0.00 | 2.87 | 2.25 | 0.11 | 0.00 | 0.03 | 0.01 |

Estimates of fixed effects derived from linear mixed models, random effect: study

Gender reference is women.

^1^ Partial eta squared derived from GLM including study as covariate.

**Age of Onset**

| Parameter | Estimate | Std. Error | df | t | Sig. | 95% Confidence Interval | |  |
| --- | --- | --- | --- | --- | --- | --- | --- | --- |
|  |  |  |  |  |  | Lower Bound | Upper Bound | **Partial Eta Squared ^1^** |
| Intercept | -1.96 | 0.42 | 378.45 | -4.63 | <.001 | -2.79 | -1.13 | 0.05 |
| age | 0.05 | 0.01 | 368.71 | 8.11 | <.001 | 0.04 | 0.06 | 0.11 |
| education | -0.07 | 0.01 | 326.31 | -5.40 | <.001 | -0.10 | -0.04 | 0.05 |
| Gender [men] | -0.19 | 0.08 | 463.74 | -2.40 | 0.02 | -0.35 | -0.04 | 0.01 |
| Age of onset | 0.00 | 0.00 | 449.51 | -0.47 | 0.64 | -0.01 | 0.00 | 0.00 |

Estimates of fixed effects derived from linear mixed models, random effect: study

Gender reference is women.

^1^ Partial eta squared derived from GLM including study as covariate.

**Depression Severity**

| Parameter | Estimate | Std. Error | df | t | Sig. | 95% Confidence Interval | |  |
| --- | --- | --- | --- | --- | --- | --- | --- | --- |
|  |  |  |  |  |  | Lower Bound | Upper Bound | **Partial Eta Squared ^1^** |
| Intercept | -2.02 | 0.43 | 335.01 | -4.69 | <.001 | -2.87 | -1.17 | 0.05 |
| age | 0.05 | 0.01 | 306.13 | 8.44 | <.001 | 0.04 | 0.06 | 0.11 |
| education | -0.07 | 0.01 | 345.76 | -5.23 | <.001 | -0.09 | -0.04 | 0.04 |
| Gender [men] | -0.18 | 0.08 | 488.75 | -2.34 | 0.02 | -0.34 | -0.03 | 0.01 |
| Depression [no depression] | 0.19 | 0.18 | 458.89 | 1.06 | 0.29 | -0.16 | 0.54 | 0.00 |
| Depression [mild-moderate depression] | -2.02 | 0.43 | 335.01 | -4.69 | <.001 | -2.87 | -1.17 | 0.05 |

Estimates of fixed effects derived from linear mixed models, random effect: study

Gender reference is women, Depression severity reference is severe depression (versus none/mild-moderate).

^1^ Partial eta squared derived from GLM including study as covariate.

**Lithium Use**

| Parameter | Estimate | Std. Error | df | t | Sig. | 95% Confidence Interval | |  |
| --- | --- | --- | --- | --- | --- | --- | --- | --- |
|  |  |  |  |  |  | Lower Bound | Upper Bound | **Partial Eta Squared ^1^** |
| Intercept | -2.07 | 0.41 | 471.00 | -5.00 | <.001 | -2.89 | -1.26 | 0.04 |
| age | 0.05 | 0.01 | 471.00 | 8.96 | <.001 | 0.04 | 0.06 | 0.11 |
| education | -0.06 | 0.01 | 471.00 | -4.76 | <.001 | -0.09 | -0.04 | 0.04 |
| Gender [men] | -0.19 | 0.08 | 471.00 | -2.42 | 0.02 | -0.35 | -0.04 | 0.01 |
| Lithium [no current lithium use] | 0.09 | 0.08 | 471.00 | 1.11 | 0.27 | -0.07 | 0.26 | 0.00 |

Estimates of fixed effects derived from linear mixed models, random effect: study

Gender reference is women. Lithium reference is currently using lithium.

^1^ Partial eta squared derived from GLM including study as covariate.

**Anti-psychotics Use**

| Parameter | Estimate | Std. Error | df | t | Sig. | 95% Confidence Interval | |  |
| --- | --- | --- | --- | --- | --- | --- | --- | --- |
|  |  |  |  |  |  | Lower Bound | Upper Bound | **Partial Eta Squared ^1^** |
| Intercept | -2.02 | 0.41 | 385.13 | -4.86 | <.001 | -2.83 | -1.20 | 0.05 |
| age | 0.05 | 0.01 | 295.90 | 9.27 | <.001 | 0.04 | 0.06 | 0.12 |
| education | -0.06 | 0.01 | 336.66 | -4.70 | <.001 | -0.09 | -0.04 | 0.04 |
| Gender [men] | -0.17 | 0.08 | 463.66 | -2.11 | 0.04 | -0.32 | -0.01 | 0.01 |
| Antipsychotics [no current antipsychotic use] | -0.33 | 0.08 | 431.17 | -3.88 | <.001 | -0.49 | -0.16 | 0.04 |

Estimates of fixed effects derived from linear mixed models, random effect: study

Gender reference is women. Antipsychotic reference is currently using antipsychotic medication.

^1^ Partial eta squared derived from GLM including study as covariate.

**H2c-M2: Associations Between Clinical Variables and Executive Function Within OABD TMT-B Test Completers**

**Mania Severity**

| Parameter | Estimate | Std. Error | df | t | Sig. | 95% Confidence Interval | |  |
| --- | --- | --- | --- | --- | --- | --- | --- | --- |
|  |  |  |  |  |  | Lower Bound | Upper Bound | **Partial Eta Squared ^1^** |
| Intercept | -1.03 | 0.37 | 448.00 | -2.81 | 0.01 | -1.75 | -0.31 | 0.07 |
| age | 0.03 | 0.01 | 448.00 | 5.05 | <.001 | 0.02 | 0.04 | 0.06 |
| education | -0.04 | 0.01 | 448.00 | -3.55 | <.001 | -0.06 | -0.02 | 0.02 |
| Psychomotor speed (TMT-A) | 0.47 | 0.04 | 448.00 | 12.73 | <.001 | 0.40 | 0.54 | 0.21 |
| Gender [men] | -0.21 | 0.07 | 448.00 | -3.05 | 0.00 | -0.34 | -0.07 | 0.02 |
| Mania severity (YMRS) | -1.03 | 0.37 | 448.00 | -2.81 | 0.01 | -1.75 | -0.31 | 0.07 |

Estimates of fixed effects derived from linear mixed models, random effect: study

Gender reference is women.

^1^ Partial eta squared derived from GLM including study as covariate.

**Age of Onset**

| Parameter | Estimate | Std. Error | df | t | Sig. | 95% Confidence Interval | |  |
| --- | --- | --- | --- | --- | --- | --- | --- | --- |
|  |  |  |  |  |  | Lower Bound | Upper Bound | **Partial Eta Squared ^1^** |
| Intercept | -0.91 | 0.39 | 382.49 | -2.36 | 0.02 | -1.68 | -0.15 | 0.06 |
| age | 0.02 | 0.01 | 419.53 | 4.40 | <.001 | 0.01 | 0.04 | 0.05 |
| education | -0.04 | 0.01 | 400.94 | -3.14 | 0.00 | -0.06 | -0.01 | 0.02 |
| Psychomotor speed (TMT-A) | 0.48 | 0.04 | 422.66 | 12.60 | <.001 | 0.41 | 0.56 | 0.22 |
| Gender [men] | -0.21 | 0.07 | 422.75 | -3.04 | 0.00 | -0.35 | -0.08 | 0.02 |
| Age of onset | -0.91 | 0.39 | 382.49 | -2.36 | 0.02 | -1.68 | -0.15 | 0.06 |

Estimates of fixed effects derived from linear mixed models, random effect: study

Gender reference is women.

^1^ Partial eta squared derived from GLM including study as covariate.

**Depression Severity**

| Parameter | Estimate | Std. Error | df | t | Sig. | 95% Confidence Interval | |  |
| --- | --- | --- | --- | --- | --- | --- | --- | --- |
|  |  |  |  |  |  | Lower Bound | Upper Bound | **Partial Eta Squared ^1^** |
| Intercept | -1.06 | 0.40 | 384.04 | -2.64 | 0.01 | -1.85 | -0.27 | 0.06 |
| age | 0.02 | 0.01 | 438.55 | 4.76 | <.001 | 0.01 | 0.03 | 0.05 |
| education | -0.04 | 0.01 | 415.29 | -3.12 | 0.00 | -0.06 | -0.01 | 0.02 |
| Psychomotor speed (TMT-A) | 0.48 | 0.04 | 449.45 | 12.82 | <.001 | 0.40 | 0.55 | 0.22 |
| Gender [men] | -0.19 | 0.07 | 450.93 | -2.77 | 0.01 | -0.33 | -0.06 | 0.02 |
| Depression [no depression] | -1.06 | 0.40 | 384.04 | -2.64 | 0.01 | -1.85 | -0.27 | 0.06 |
| Depression [mild-moderate depression] | 0.02 | 0.01 | 438.55 | 4.76 | <.001 | 0.01 | 0.03 | 0.05 |

Estimates of fixed effects derived from linear mixed models, random effect: study

Gender reference is women, Depression severity reference is severe depression (versus none/mild-moderate).

^1^ Partial eta squared derived from GLM including study as covariate.

**Lithium Use**

| Parameter | Estimate | Std. Error | df | t | Sig. | 95% Confidence Interval | |  |
| --- | --- | --- | --- | --- | --- | --- | --- | --- |
|  |  |  |  |  |  | Lower Bound | Upper Bound | **Partial Eta Squared ^1^** |
| Intercept | -0.97 | 0.39 | 401.64 | -2.50 | 0.01 | -1.73 | -0.21 | 0.05 |
| age | 0.02 | 0.01 | 404.56 | 4.60 | <.001 | 0.01 | 0.03 | 0.05 |
| education | -0.03 | 0.01 | 403.30 | -2.69 | 0.01 | -0.05 | -0.01 | 0.02 |
| Psychomotor speed (TMT-A) | 0.47 | 0.04 | 427.95 | 12.24 | <.001 | 0.39 | 0.54 | 0.22 |
| Gender [men] | -0.22 | 0.07 | 427.85 | -3.08 | 0.00 | -0.36 | -0.08 | 0.02 |
| Lithium [no current lithium use] | -0.97 | 0.39 | 401.64 | -2.50 | 0.01 | -1.73 | -0.21 | 0.05 |

Estimates of fixed effects derived from linear mixed models, random effect: study

Gender reference is women. Lithium reference is currently using lithium.

^1^ Partial eta squared derived from GLM including study as covariate.

**Anti-psychotics Use**

| Parameter | Estimate | Std. Error | df | t | Sig. | 95% Confidence Interval | |  |
| --- | --- | --- | --- | --- | --- | --- | --- | --- |
|  |  |  |  |  |  | Lower Bound | Upper Bound | **Partial Eta Squared ^1^** |
| Intercept | -0.95 | 0.39 | 399.48 | -2.44 | 0.02 | -1.72 | -0.18 | 0.06 |
| age | 0.03 | 0.01 | 417.25 | 5.02 | <.001 | 0.02 | 0.04 | 0.05 |
| education | -0.03 | 0.01 | 409.25 | -2.66 | 0.01 | -0.05 | -0.01 | 0.02 |
| Psychomotor speed (TMT-A) | 0.46 | 0.04 | 423.91 | 11.83 | <.001 | 0.38 | 0.54 | 0.20 |
| Gender [men] | -0.21 | 0.07 | 423.97 | -2.89 | 0.00 | -0.35 | -0.07 | 0.02 |
| Antipsychotics [no current antipsychotic use] | -0.95 | 0.39 | 399.48 | -2.44 | 0.02 | -1.72 | -0.18 | 0.06 |

Estimates of fixed effects derived from linear mixed models, random effect: study

Gender reference is women. Antipsychotic reference is currently using antipsychotic medication.

^1^ Partial eta squared derived from GLM including study as covariate.

**H2c-M3: Associations Between Clinical Variables and Executive Function Within OABD TMT-B Test Completers**

**Mania Severity**

| Parameter | Estimate | Std. Error | df | t | Sig. | 95% Confidence Interval | |  |
| --- | --- | --- | --- | --- | --- | --- | --- | --- |
|  |  |  |  |  |  | Lower Bound | Upper Bound | **Partial Eta Squared ^1^** |
| Intercept | -2.18 | 0.50 | 203.15 | -4.39 | <.001 | -3.15 | -1.20 | 0.06 |
| age | 0.05 | 0.01 | 258.32 | 6.92 | <.001 | 0.03 | 0.06 | 0.12 |
| education | -0.09 | 0.01 | 269.57 | -5.72 | <.001 | -0.12 | -0.06 | 0.07 |
| Gender [men] | -0.21 | 0.09 | 331.51 | -2.28 | 0.02 | -0.39 | -0.03 | 0.01 |
| Occupation status [not employed] | 0.38 | 0.11 | 331.70 | 3.42 | <.001 | 0.16 | 0.59 | 0.04 |
| Mania severity (YMRS) | -2.18 | 0.50 | 203.15 | -4.39 | <.001 | -3.15 | -1.20 | 0.06 |

Estimates of fixed effects derived from linear mixed models, random effect: study

Gender reference is women. Occupation reference is being employed

^1^ Partial eta squared derived from GLM including study as covariate.

**Age of Onset**

| Parameter | Estimate | Std. Error | df | t | Sig. | 95% Confidence Interval | |  |
| --- | --- | --- | --- | --- | --- | --- | --- | --- |
|  |  |  |  |  |  | Lower Bound | Upper Bound | **Partial Eta Squared ^1^** |
| Intercept | -2.25 | 0.54 | 234.36 | -4.18 | <.001 | -3.31 | -1.19 | 0.05 |
| age | 0.06 | 0.01 | 269.40 | 6.65 | <.001 | 0.04 | 0.07 | 0.12 |
| education | -0.09 | 0.02 | 275.64 | -5.84 | <.001 | -0.12 | -0.06 | 0.09 |
| Gender [men] | -0.18 | 0.10 | 306.92 | -1.91 | 0.06 | -0.37 | 0.01 | 0.01 |
| Occupation status [not employed] | 0.34 | 0.12 | 306.08 | 2.94 | 0.00 | 0.11 | 0.57 | 0.03 |
| Age of onset | -2.25 | 0.54 | 234.36 | -4.18 | <.001 | -3.31 | -1.19 | 0.05 |

Estimates of fixed effects derived from linear mixed models, random effect: study

Gender reference is women. Occupation reference is being employed.

^1^ Partial eta squared derived from GLM including study as covariate.

**Depression Severity**

| Parameter | Estimate | Std. Error | df | t | Sig. | 95% Confidence Interval | |  |
| --- | --- | --- | --- | --- | --- | --- | --- | --- |
|  |  |  |  |  |  | Lower Bound | Upper Bound | **Partial Eta Squared ^1^** |
| Intercept | -2.14 | 0.52 | 213.55 | -4.10 | <.001 | -3.16 | -1.11 | 0.04 |
| age | 0.05 | 0.01 | 266.07 | 6.41 | <.001 | 0.03 | 0.06 | 0.10 |
| education | -0.09 | 0.02 | 298.71 | -5.62 | <.001 | -0.12 | -0.06 | 0.07 |
| Gender [men] | -0.18 | 0.09 | 332.88 | -1.99 | 0.05 | -0.36 | 0.00 | 0.01 |
| Occupation status [not employed] | 0.40 | 0.11 | 332.56 | 3.63 | <.001 | 0.18 | 0.62 | 0.04 |
| Depression [no depression] | 0.29 | 0.19 | 332.74 | 1.55 | 0.12 | -0.08 | 0.65 | 0.01 |
| Depression [mild-moderate depression] | 0.12 | 0.18 | 331.01 | 0.68 | 0.50 | -0.24 | 0.48 | 0.01 |

Estimates of fixed effects derived from linear mixed models, random effect: study

Gender reference is women. Occupation reference is being employed. Depression severity reference is severe depression (versus none/mild-moderate).

^1^ Partial eta squared derived from GLM including study as covariate.

**Lithium Use**

| Parameter | Estimate | Std. Error | df | t | Sig. | 95% Confidence Interval | |  |
| --- | --- | --- | --- | --- | --- | --- | --- | --- |
|  |  |  |  |  |  | Lower Bound | Upper Bound | **Partial Eta Squared ^1^** |
| Intercept | -2.26 | 0.51 | 311.00 | -4.41 | <.001 | -3.26 | -1.25 | 0.04 |
| age | 0.05 | 0.01 | 311.00 | 7.12 | <.001 | 0.04 | 0.06 | 0.10 |
| education | -0.08 | 0.02 | 311.00 | -5.13 | <.001 | -0.11 | -0.05 | 0.07 |
| Gender [men] | -0.19 | 0.10 | 311.00 | -1.99 | 0.05 | -0.38 | 0.00 | 0.01 |
| Occupation status [not employed] | 0.41 | 0.11 | 311.00 | 3.63 | <.001 | 0.19 | 0.63 | 0.04 |
| Lithium [no current lithium use] | 0.06 | 0.11 | 311.00 | 0.51 | 0.61 | -0.16 | 0.28 | 0.00 |

Estimates of fixed effects derived from linear mixed models, random effect: study

Gender reference is women. Occupation reference is being employed. Lithium reference is currently using lithium.

^1^ Partial eta squared derived from GLM including study as covariate.

**Anti-psychotics Use**

| Parameter | Estimate | Std. Error | df | t | Sig. | 95% Confidence Interval | |  |
| --- | --- | --- | --- | --- | --- | --- | --- | --- |
|  |  |  |  |  |  | Lower Bound | Upper Bound | **Partial Eta Squared ^1^** |
| Intercept | -2.21 | 0.52 | 186.39 | -4.26 | <.001 | -3.23 | -1.19 | 0.04 |
| age | 0.05 | 0.01 | 174.96 | 7.15 | <.001 | 0.04 | 0.07 | 0.11 |
| education | -0.08 | 0.02 | 291.88 | -4.97 | <.001 | -0.11 | -0.05 | 0.07 |
| Gender [men] | -0.17 | 0.09 | 304.31 | -1.82 | 0.07 | -0.36 | 0.01 | 0.01 |
| Occupation status [not employed] | 0.38 | 0.11 | 305.87 | 3.34 | <.001 | 0.16 | 0.60 | 0.03 |
| Antipsychotics [no current antipsychotic use] | -0.23 | 0.10 | 294.24 | -2.28 | 0.02 | -0.43 | -0.03 | 0.02 |

Estimates of fixed effects derived from linear mixed models, random effect: study

Gender reference is women. Occupation reference is being employed. Antipsychotic reference is currently using antipsychotic medication.

^1^ Partial eta squared derived from GLM including study as covariate.

**H2c-M4: Associations Between Clinical Variables and Executive Function Within OABD TMT-B Test Completers**

**Mania Severity**

| Parameter | Estimate | Std. Error | df | t | Sig. | 95% Confidence Interval | |  |
| --- | --- | --- | --- | --- | --- | --- | --- | --- |
|  |  |  |  |  |  | Lower Bound | Upper Bound | **Partial Eta Squared ^1^** |
| Intercept | -1.22 | 0.46 | 188.69 | -2.63 | 0.01 | -2.13 | -0.31 | 0.07 |
| age | 0.03 | 0.01 | 295.03 | 4.28 | <.001 | 0.02 | 0.04 | 0.06 |
| education | -0.06 | 0.01 | 242.60 | -4.23 | <.001 | -0.08 | -0.03 | 0.05 |
| Psychomotor speed (TMT-A) | 0.42 | 0.04 | 295.95 | 9.78 | <.001 | 0.34 | 0.51 | 0.21 |
| Gender [men] | -0.23 | 0.08 | 295.47 | -2.83 | 0.01 | -0.39 | -0.07 | 0.03 |
| Occupation status [not employed] | 0.35 | 0.10 | 295.75 | 3.49 | <.001 | 0.15 | 0.55 | 0.05 |
| Mania severity (YMRS) | -1.22 | 0.46 | 188.69 | -2.63 | 0.01 | -2.13 | -0.31 | 0.07 |

Estimates of fixed effects derived from linear mixed models, random effect: study

Gender reference is women. Occupation reference is being employed

^1^ Partial eta squared derived from GLM including study as covariate.

**Age of Onset**

| Parameter | Estimate | Std. Error | df | t | Sig. | 95% Confidence Interval | |  |
| --- | --- | --- | --- | --- | --- | --- | --- | --- |
|  |  |  |  |  |  | Lower Bound | Upper Bound | **Partial Eta Squared ^1^** |
| Intercept | -0.99 | 0.50 | 230.49 | -1.98 | 0.05 | -1.98 | 0.00 | 0.06 |
| age | 0.03 | 0.01 | 271.85 | 3.49 | <.001 | 0.01 | 0.04 | 0.04 |
| education | -0.06 | 0.01 | 269.14 | -4.01 | <.001 | -0.08 | -0.03 | 0.05 |
| Psychomotor speed (TMT-A) | 0.45 | 0.05 | 270.64 | 10.05 | <.001 | 0.36 | 0.54 | 0.24 |
| Gender [men] | -0.23 | 0.08 | 270.15 | -2.79 | 0.01 | -0.40 | -0.07 | 0.03 |
| Occupation status [not employed] | 0.33 | 0.10 | 271.80 | 3.12 | 0.00 | 0.12 | 0.53 | 0.05 |
| Age of onset | -0.99 | 0.50 | 230.49 | -1.98 | 0.05 | -1.98 | 0.00 | 0.06 |

Estimates of fixed effects derived from linear mixed models, random effect: study

Gender reference is women. Occupation reference is being employed

^1^ Partial eta squared derived from GLM including study as covariate.

**Depression Severity**

| Parameter | Estimate | Std. Error | df | t | Sig. | 95% Confidence Interval | |  |
| --- | --- | --- | --- | --- | --- | --- | --- | --- |
|  |  |  |  |  |  | Lower Bound | Upper Bound | **Partial Eta Squared ^1^** |
| Intercept | -1.15 | 0.49 | 230.18 | -2.36 | 0.02 | -2.11 | -0.19 | 0.05 |
| age | 0.03 | 0.01 | 297.96 | 3.79 | <.001 | 0.01 | 0.04 | 0.04 |
| education | -0.05 | 0.01 | 289.46 | -4.01 | <.001 | -0.08 | -0.03 | 0.05 |
| Psychomotor speed (TMT-A) | 0.44 | 0.04 | 295.89 | 10.05 | <.001 | 0.35 | 0.52 | 0.23 |
| Gender [men] | -0.21 | 0.08 | 297.06 | -2.55 | 0.01 | -0.37 | -0.05 | 0.02 |
| Occupation status [not employed] | 0.36 | 0.10 | 296.47 | 3.57 | <.001 | 0.16 | 0.55 | 0.01 |
| Depression [no depression] | -1.15 | 0.49 | 230.18 | -2.36 | 0.02 | -2.11 | -0.19 | 0.05 |
| Depression [mild-moderate depression] | 0.03 | 0.01 | 297.96 | 3.79 | <.001 | 0.01 | 0.04 | 0.04 |

Estimates of fixed effects derived from linear mixed models, random effect: study

Gender reference is women. Occupation reference is being employed. Depression severity reference is severe depression (versus none/mild-moderate).

^1^ Partial eta squared derived from GLM including study as covariate.

**Lithium Use**

| Parameter | Estimate | Std. Error | df | t | Sig. | 95% Confidence Interval | |  |
| --- | --- | --- | --- | --- | --- | --- | --- | --- |
|  |  |  |  |  |  | Lower Bound | Upper Bound | **Partial Eta Squared ^1^** |
| Intercept | -1.10 | 0.48 | 208.64 | -2.28 | 0.02 | -2.06 | -0.15 | 0.05 |
| age | 0.03 | 0.01 | 263.68 | 3.81 | <.001 | 0.01 | 0.04 | 0.04 |
| education | -0.05 | 0.01 | 272.24 | -3.51 | <.001 | -0.08 | -0.02 | 0.04 |
| Psychomotor speed (TMT-A) | 0.43 | 0.05 | 274.79 | 9.54 | <.001 | 0.34 | 0.52 | 0.23 |
| Gender [men] | -0.25 | 0.09 | 274.64 | -2.98 | 0.00 | -0.42 | -0.09 | 0.03 |
| Occupation status [not employed] | 0.38 | 0.10 | 274.96 | 3.71 | <.001 | 0.18 | 0.59 | 0.06 |
| Lithium [no current lithium use] | -1.10 | 0.48 | 208.64 | -2.28 | 0.02 | -2.06 | -0.15 | 0.05 |

Estimates of fixed effects derived from linear mixed models, random effect: study

Gender reference is women. Occupation reference is being employed. Lithium reference is currently using lithium.

^1^ Partial eta squared derived from GLM including study as covariate.

**Anti-psychotics Use**

| Parameter | Estimate | Std. Error | df | t | Sig. | 95% Confidence Interval | |  |
| --- | --- | --- | --- | --- | --- | --- | --- | --- |
|  |  |  |  |  |  | Lower Bound | Upper Bound | **Partial Eta Squared ^1^** |
| Intercept | -1.07 | 0.50 | 234.48 | -2.15 | 0.03 | -2.05 | -0.09 | 0.05 |
| age | 0.03 | 0.01 | 268.87 | 4.04 | <.001 | 0.01 | 0.04 | 0.05 |
| education | -0.05 | 0.01 | 269.60 | -3.41 | <.001 | -0.08 | -0.02 | 0.04 |
| Psychomotor speed (TMT-A) | 0.42 | 0.05 | 270.70 | 9.29 | <.001 | 0.33 | 0.51 | 0.22 |
| Gender [men] | -0.23 | 0.09 | 270.85 | -2.72 | 0.01 | -0.40 | -0.06 | 0.03 |
| Occupation status [not employed] | 0.35 | 0.10 | 270.71 | 3.38 | <.001 | 0.15 | 0.56 | 0.05 |
| Antipsychotics [no current antipsychotic use] | -0.13 | 0.09 | 269.47 | -1.40 | 0.16 | -0.31 | 0.05 | 0.01 |

Estimates of fixed effects derived from linear mixed models, random effect: study

Gender reference is women. Occupation reference is being employed. Antipsychotic reference is currently using antipsychotic medication.

^1^ Partial eta squared derived from GLM including study as covariate.

**H2d-M1: Associations Between Clinical Variables and TMT-B Test Completion in OABD Participants**

**Mania Severity**

| **Parameter** | **Estimate** | **Std. Error** | **df** | **t** | **Sig.** | **95% Confidence Interval** | |  |
| --- | --- | --- | --- | --- | --- | --- | --- | --- |
|  |  |  |  |  |  | Lower Bound | Upper Bound | **Partial Eta Squared ^1^** |
| Intercept | 5.82 | 3.09 | 42.37 | 1.89 | 0.07 | -0.41 | 12.05 | 0.08 |
| age | 0.00 | 0.03 | 562.67 | 0.15 | 0.88 | -0.06 | 0.06 | 0.02 |
| education | -0.04 | 0.07 | 561.16 | -0.56 | 0.58 | -0.17 | 0.10 | 0.04 |
| Gender [men] | 0.20 | 0.42 | 560.39 | 0.49 | 0.63 | -0.62 | 1.03 | 0.00 |
| Executive functioning (TMT-B test non-completer) | 2.24 | 0.62 | 559.59 | 3.59 | <.001 | 1.02 | 3.47 | 0.02 |

Estimates of fixed effects derived from linear mixed models, random effect: study

Gender reference is women, test completion reference is test completion of TMT-B.

^1^ Partial eta squared derived from GLM including study as covariate.

**Age of Onset**

| **Parameter** | **Estimate** | **Std. Error** | **df** | **t** | **Sig.** | **95% Confidence Interval** | |  |
| --- | --- | --- | --- | --- | --- | --- | --- | --- |
|  |  |  |  |  |  | **Lower Bound** | **Upper Bound** | **Partial Eta Squared ^1^** |
| Intercept | -21.83 | 6.94 | 398.08 | -3.15 | 0.00 | -35.47 | -8.19 | 0.07 |
| age | 0.81 | 0.09 | 440.64 | 8.73 | <.001 | 0.63 | 0.99 | 0.01 |
| education | 0.07 | 0.21 | 511.78 | 0.33 | 0.74 | -0.34 | 0.48 | 0.05 |
| Gender [men] | -0.60 | 1.30 | 539.27 | -0.46 | 0.65 | -3.15 | 1.96 | 0.00 |
| Executive functioning (TMT-B test non-completer) | 1.56 | 1.96 | 537.65 | 0.80 | 0.43 | -2.29 | 5.41 | 0.00 |

Estimates of fixed effects derived from linear mixed models, random effect: study

Gender reference is women, test completion reference is test completion of TMT-B.

^1^ Partial eta squared derived from GLM including study as covariate.

**Depression Severity**

| Parameter | Estimate | Std. Error | df | t | Sig. | 95% Confidence Interval | |  |
| --- | --- | --- | --- | --- | --- | --- | --- | --- |
|  |  |  |  |  |  | Lower Bound | Upper Bound | **Partial Eta Squared ^1^** |
| Intercept | 1.14 | 0.27 | 178.75 | 4.29 | <.001 | 0.61 | 1.66 | 0.07 |
| age | -0.01 | 0.00 | 572.53 | -2.26 | 0.02 | -0.01 | 0.00 | 0.02 |
| education | -0.01 | 0.01 | 569.32 | -1.11 | 0.27 | -0.02 | 0.01 | 0.05 |
| Gender [men] | -0.03 | 0.04 | 567.08 | -0.66 | 0.51 | -0.12 | 0.06 | 0.00 |
| Executive functioning (TMT-B test non-completer) | 0.02 | 0.07 | 564.01 | 0.37 | 0.72 | -0.11 | 0.16 | 0.00 |

Estimates of fixed effects derived from linear mixed models, random effect: study

Gender reference is women, test completion reference is completion of TMT-B.

^1^ Partial eta squared derived from GLM including study as covariate

**Lithium Use**

| Parameter | Estimate | Std. Error | df | t | Sig. | 95% Confidence Interval | |  |
| --- | --- | --- | --- | --- | --- | --- | --- | --- |
|  |  |  |  |  |  | Lower Bound | Upper Bound | **Partial Eta Squared ^1^** |
| Intercept | 0.55 | 0.21 | 128.21 | 2.62 | 0.01 | 0.13 | 0.96 | 0.07 |
| age | 0.00 | 0.00 | 543.78 | -1.31 | 0.19 | -0.01 | 0.00 | 0.02 |
| education | 0.00 | 0.01 | 539.61 | 0.53 | 0.60 | -0.01 | 0.01 | 0.05 |
| Gender [men] | -0.03 | 0.03 | 539.15 | -0.93 | 0.35 | -0.10 | 0.04 | 0.00 |
| Executive functioning (TMT-B test non-completer) | 0.03 | 0.05 | 536.67 | 0.56 | 0.58 | -0.07 | 0.13 | 0.00 |

Estimates of fixed effects derived from linear mixed models, random effect: study

Gender reference is women, test completion reference is test completion of TMT-B.

^1^ Partial eta squared derived from GLM including study as covariate

**Anti-psychotics Use**

| Parameter | Estimate | Std. Error | df | t | Sig. | 95% Confidence Interval | |  |
| --- | --- | --- | --- | --- | --- | --- | --- | --- |
|  |  |  |  |  |  | Lower Bound | Upper Bound | **Partial Eta Squared ^1^** |
| Intercept | 0.92 | 0.22 | 445.07 | 4.24 | <.001 | 0.50 | 1.35 | 0.07 |
| age | -0.01 | 0.00 | 480.73 | -2.18 | 0.03 | -0.01 | 0.00 | 0.02 |
| education | -0.01 | 0.01 | 536.92 | -1.33 | 0.18 | -0.02 | 0.00 | 0.04 |
| Gender [men] | -0.05 | 0.04 | 540.98 | -1.12 | 0.26 | -0.12 | 0.03 | 0.00 |
| Executive functioning (TMT-B test non-completer) | 0.10 | 0.06 | 537.86 | 1.58 | 0.11 | -0.02 | 0.22 | 0.01 |

Estimates of fixed effects derived from linear mixed models, random effect: study

Gender reference is women, test completion reference is test completion of TMT-B.

^1^ Partial eta squared derived from GLM including study as covariate

**H2d-M2: Associations Between Clinical Variables and TMT-B Test Completion in OABD Participants**

**Mania Severity**

| Parameter | Estimate | Std. Error | df | t | Sig. | 95% Confidence Interval | |  |
| --- | --- | --- | --- | --- | --- | --- | --- | --- |
|  |  |  |  |  |  | Lower Bound | Upper Bound | **Partial Eta Squared ^1^** |
| Intercept | 6.43 | 3.47 | 25.77 | 1.85 | 0.08 | -0.72 | 13.57 | 0.09 |
| age | 0.00 | 0.03 | 517.29 | 0.02 | 0.98 | -0.06 | 0.06 | 0.00 |
| education | -0.04 | 0.07 | 517.50 | -0.54 | 0.59 | -0.18 | 0.10 | 0.01 |
| Gender [men] | 0.15 | 0.43 | 516.73 | 0.35 | 0.72 | -0.70 | 1.01 | 0.00 |
| Psychomotor speed (TMT A) | 0.00 | 0.01 | 516.31 | 0.09 | 0.93 | -0.01 | 0.01 | 0.27 |
| Executive functioning (TMT-B test non-completer) | 6.43 | 3.47 | 25.77 | 1.85 | 0.08 | -0.72 | 13.57 | 0.09 |

Estimates of fixed effects derived from linear mixed models, random effect: study

Gender reference is women, test completion reference is test completion of TMT-B.

^1^ Partial eta squared derived from GLM including study as covariate

**Age of Onset**

| Parameter | Estimate | Std. Error | df | t | Sig. | 95% Confidence Interval | |  |
| --- | --- | --- | --- | --- | --- | --- | --- | --- |
|  |  |  |  |  |  | Lower Bound | Upper Bound | **Partial Eta Squared ^1^** |
| Intercept | -18.06 | 7.43 | 420.86 | -2.43 | 0.02 | -32.67 | -3.46 | 0.08 |
| age | 0.72 | 0.10 | 479.17 | 7.07 | <.001 | 0.52 | 0.92 | 0.00 |
| education | 0.16 | 0.22 | 456.30 | 0.71 | 0.48 | -0.28 | 0.59 | 0.02 |
| Gender [men] | -0.43 | 1.38 | 494.52 | -0.31 | 0.76 | -3.14 | 2.28 | 0.00 |
| Psychomotor speed (TMT A) | 0.04 | 0.02 | 493.20 | 1.64 | 0.10 | -0.01 | 0.08 | 0.26 |
| Executive functioning (TMT-B test non-completer) | -18.06 | 7.43 | 420.86 | -2.43 | 0.02 | -32.67 | -3.46 | 0.08 |

Estimates of fixed effects derived from linear mixed models, random effect: study

Gender reference is women, test completion reference is test completion of TMT-B.

^1^ Partial eta squared derived from GLM including study as covariate

**Depression Severity**

| Parameter | Estimate | Std. Error | df | t | Sig. | 95% Confidence Interval | |  |
| --- | --- | --- | --- | --- | --- | --- | --- | --- |
|  |  |  |  |  |  | Lower Bound | Upper Bound | **Partial Eta Squared ^1^** |
| Intercept | 1.18 | 0.28 | 165.50 | 4.26 | <.001 | 0.63 | 1.73 | 0.08 |
| age | -0.01 | 0.00 | 525.67 | -2.05 | 0.04 | -0.01 | 0.00 | 0.00 |
| education | -0.01 | 0.01 | 526.00 | -1.29 | 0.20 | -0.02 | 0.01 | 0.02 |
| Gender [men] | -0.03 | 0.05 | 522.82 | -0.65 | 0.52 | -0.12 | 0.06 | 0.00 |
| Psychomotor speed (TMT A) | 0.00 | 0.00 | 520.54 | -0.58 | 0.56 | 0.00 | 0.00 | 0.28 |
| Executive functioning (TMT-B test non-completer) | 1.18 | 0.28 | 165.50 | 4.26 | <.001 | 0.63 | 1.73 | 0.08 |

Estimates of fixed effects derived from linear mixed models, random effect: study

Gender reference is women, test completion reference is test completion of TMT-B.

^1^ Partial eta squared derived from GLM including study as covariate

**Lithium Use**

| Parameter | Estimate | Std. Error | df | t | Sig. | 95% Confidence Interval | |  |
| --- | --- | --- | --- | --- | --- | --- | --- | --- |
|  |  |  |  |  |  | Lower Bound | Upper Bound | **Partial Eta Squared ^1^** |
| Intercept | 0.68 | 0.22 | 95.10 | 3.04 | 0.00 | 0.24 | 1.13 | 0.09 |
| age | 0.00 | 0.00 | 495.78 | -1.22 | 0.22 | -0.01 | 0.00 | 0.00 |
| education | 0.00 | 0.01 | 494.77 | 0.13 | 0.90 | -0.01 | 0.01 | 0.04 |
| Gender [men] | -0.03 | 0.04 | 493.97 | -0.92 | 0.36 | -0.11 | 0.04 | 0.00 |
| Psychomotor speed (TMT A) | 0.00 | 0.00 | 492.19 | -0.94 | 0.35 | 0.00 | 0.00 | 0.27 |
| Executive functioning (TMT-B test non-completer) | 0.68 | 0.22 | 95.10 | 3.04 | 0.00 | 0.24 | 1.13 | 0.09 |

Estimates of fixed effects derived from linear mixed models, random effect: study

Gender reference is women, test completion reference is test completion of TMT-B.

^1^ Partial eta squared derived from GLM including study as covariate

**Anti-psychotics Use**

| Parameter | Estimate | Std. Error | df | t | Sig. | 95% Confidence Interval | |  |
| --- | --- | --- | --- | --- | --- | --- | --- | --- |
|  |  |  |  |  |  | Lower Bound | Upper Bound | **Partial Eta Squared ^1^** |
| Intercept | 0.85 | 0.23 | 412.35 | 3.74 | <.001 | 0.40 | 1.29 | 0.09 |
| age | -0.01 | 0.00 | 492.13 | -2.30 | 0.02 | -0.01 | 0.00 | 0.00 |
| education | -0.01 | 0.01 | 493.72 | -1.10 | 0.27 | -0.02 | 0.01 | 0.02 |
| Gender [men] | -0.04 | 0.04 | 494.36 | -0.97 | 0.33 | -0.12 | 0.04 | 0.00 |
| Psychomotor speed (TMT A) | 0.00 | 0.00 | 490.80 | 2.54 | 0.01 | 0.00 | 0.00 | 0.27 |
| Executive functioning (TMT-B test non-completer) | 0.85 | 0.23 | 412.35 | 3.74 | <.001 | 0.40 | 1.29 | 0.09 |

Estimates of fixed effects derived from linear mixed models, random effect: study

Gender reference is women, test completion reference is test completion of TMT-B.

^1^ Partial eta squared derived from GLM including study as covariate

**H2d-M3: Associations Between Clinical Variables and TMT-B Test Completion in OABD Participants**

**Mania Severity**

| Parameter | Estimate | Std. Error | df | t | Sig. | 95% Confidence Interval | |  |
| --- | --- | --- | --- | --- | --- | --- | --- | --- |
|  |  |  |  |  |  | Lower Bound | Upper Bound | **Partial Eta Squared ^1^** |
| Intercept | 7.88 | 4.26 | 42.32 | 1.85 | 0.07 | -0.72 | 16.48 | 0.09 |
| age | 0.00 | 0.05 | 380.30 | -0.07 | 0.94 | -0.10 | 0.09 | 0.02 |
| education | -0.06 | 0.10 | 378.91 | -0.59 | 0.55 | -0.25 | 0.14 | 0.03 |
| Gender [men] | 0.32 | 0.60 | 378.25 | 0.52 | 0.60 | -0.87 | 1.51 | 0.00 |
| Occupation status [not employed] | 0.61 | 0.75 | 377.47 | 0.81 | 0.42 | -0.87 | 2.08 | 0.00 |
| Executive functioning (TMT-B test non-completer) | 7.88 | 4.26 | 42.32 | 1.85 | 0.07 | -0.72 | 16.48 | 0.09 |

Estimates of fixed effects derived from linear mixed models, random effect: study

Gender reference is women, test completion reference is test completion of TMT-B, occupation reference is being employed.

^1^ Partial eta squared derived from GLM including study as covariate

**Age of Onset**

| Parameter | Estimate | Std. Error | df | t | Sig. | 95% Confidence Interval | |  |
| --- | --- | --- | --- | --- | --- | --- | --- | --- |
|  |  |  |  |  |  | Lower Bound | Upper Bound | **Partial Eta Squared ^1^** |
| Intercept | -30.25 | 8.64 | 122.57 | -3.50 | <.001 | -47.36 | -13.14 | 0.07 |
| age | 0.89 | 0.12 | 138.02 | 7.12 | <.001 | 0.64 | 1.14 | 0.01 |
| education | 0.20 | 0.26 | 208.30 | 0.77 | 0.44 | -0.31 | 0.71 | 0.05 |
| Gender [men] | -0.80 | 1.63 | 353.88 | -0.49 | 0.62 | -4.00 | 2.40 | 0.00 |
| Occupation status [not employed] | 0.10 | 2.06 | 354.58 | 0.05 | 0.96 | -3.96 | 4.16 | 0.00 |
| Executive functioning (TMT-B test non-completer) | -30.25 | 8.64 | 122.57 | -3.50 | <.001 | -47.36 | -13.14 | 0.07 |

Estimates of fixed effects derived from linear mixed models, random effect: study

Gender reference is women, test completion reference is test completion of TMT-B, occupation reference is being employed.

^1^ Partial eta squared derived from GLM including study as covariate

**Depression Severity**

| Parameter | Estimate | Std. Error | df | t | Sig. | 95% Confidence Interval | |  |
| --- | --- | --- | --- | --- | --- | --- | --- | --- |
|  |  |  |  |  |  | Lower Bound | Upper Bound | **Partial Eta Squared ^1^** |
| Intercept | 1.73 | 0.35 | 177.23 | 4.99 | <.001 | 1.05 | 2.42 | 0.08 |
| age | -0.01 | 0.00 | 386.91 | -3.12 | 0.00 | -0.02 | -0.01 | 0.02 |
| education | -0.01 | 0.01 | 385.59 | -1.33 | 0.19 | -0.03 | 0.01 | 0.04 |
| Gender [men] | -0.05 | 0.06 | 383.73 | -0.78 | 0.44 | -0.16 | 0.07 | 0.00 |
| Occupation status [not employed] | 0.10 | 0.07 | 380.59 | 1.40 | 0.16 | -0.04 | 0.24 | 0.00 |
| Executive functioning (TMT-B test non-completer) | 1.73 | 0.35 | 177.23 | 4.99 | <.001 | 1.05 | 2.42 | 0.08 |

Estimates of fixed effects derived from linear mixed models, random effect: study

Gender reference is women, test completion reference is test completion of TMT-B, occupation reference is being employed.

^1^ Partial eta squared derived from GLM including study as covariate

**Lithium Use**

| **Estimates of Fixed Effects^a^** | | | | | | | |  |
| --- | --- | --- | --- | --- | --- | --- | --- | --- |
| Parameter | Estimate | Std. Error | df | t | Sig. | 95% Confidence Interval | |  |
|  |  |  |  |  |  | Lower Bound | Upper Bound | **Partial Eta Squared ^1^** |
| Intercept | 0.31 | 0.23 | 142.93 | 1.33 | 0.19 | -0.15 | 0.78 | 0.07 |
| age | 0.00 | 0.00 | 358.98 | -0.08 | 0.94 | -0.01 | 0.01 | 0.02 |
| education | 0.00 | 0.01 | 356.36 | -0.26 | 0.79 | -0.01 | 0.01 | 0.04 |
| Gender [men] | -0.07 | 0.04 | 356.18 | -1.84 | 0.07 | -0.15 | 0.01 | 0.00 |
| Occupation status [not employed] | 0.01 | 0.05 | 354.01 | 0.25 | 0.81 | -0.08 | 0.10 | 0.00 |
| Executive functioning (TMT-B test non-completer) | 0.31 | 0.23 | 142.93 | 1.33 | 0.19 | -0.15 | 0.78 | 0.07 |

Estimates of fixed effects derived from linear mixed models, random effect: study

Gender reference is women, test completion reference is test completion of TMT-B, occupation reference is being employed.

^1^ Partial eta squared derived from GLM including study as covariate

**Anti-psychotics Use**

| **Estimates of Fixed Effects^a^** | | | | | | | |  |
| --- | --- | --- | --- | --- | --- | --- | --- | --- |
| Parameter | Estimate | Std. Error | df | t | Sig. | 95% Confidence Interval | |  |
|  |  |  |  |  |  | Lower Bound | Upper Bound | **Partial Eta Squared ^1^** |
| Intercept | 0.88 | 0.28 | 270.14 | 3.15 | 0.00 | 0.33 | 1.44 | 0.07 |
| age | -0.01 | 0.00 | 281.80 | -1.61 | 0.11 | -0.01 | 0.00 | 0.02 |
| education | -0.01 | 0.01 | 351.10 | -1.66 | 0.10 | -0.03 | 0.00 | 0.04 |
| Gender [men] | -0.04 | 0.05 | 354.26 | -0.73 | 0.47 | -0.14 | 0.06 | 0.00 |
| Occupation status [not employed] | 0.15 | 0.06 | 353.27 | 2.35 | 0.02 | 0.02 | 0.27 | 0.00 |
| Executive functioning (TMT-B test non-completer) | 0.88 | 0.28 | 270.14 | 3.15 | 0.00 | 0.33 | 1.44 | 0.07 |

Estimates of fixed effects derived from linear mixed models, random effect: study

Gender reference is women, test completion reference is test completion of TMT-B, occupation reference is being employed.

^1^ Partial eta squared derived from GLM including study as covariate

**H2d-M4: Associations Between Clinical Variables and TMT-B Test Completion in OABD Participants**

**Mania Severity**

| Parameter | Estimate | Std. Error | df | t | Sig. | 95% Confidence Interval | |  |
| --- | --- | --- | --- | --- | --- | --- | --- | --- |
|  |  |  |  |  |  | Lower Bound | Upper Bound | **Partial Eta Squared ^1^** |
| Intercept | 9.44 | 4.94 | 20.77 | 1.91 | 0.07 | -0.85 | 19.72 | 0.10 |
| age | -0.01 | 0.05 | 340.41 | -0.26 | 0.79 | -0.11 | 0.09 | 0.00 |
| education | -0.05 | 0.10 | 341.20 | -0.50 | 0.62 | -0.26 | 0.15 | 0.01 |
| Gender [men] | 0.22 | 0.64 | 340.33 | 0.34 | 0.74 | -1.04 | 1.47 | 0.00 |
| Psychomotor speed (TMT A) | 0.00 | 0.01 | 340.28 | 0.03 | 0.97 | -0.02 | 0.02 | 0.29 |
| Occupation status [not employed] | 0.75 | 0.81 | 340.35 | 0.92 | 0.36 | -0.85 | 2.34 | 0.00 |
| Executive functioning (TMT-B test non-completer) | 3.07 | 1.12 | 340.37 | 2.73 | 0.01 | 0.86 | 5.28 | 0.02 |

Estimates of fixed effects derived from linear mixed models, random effect: study

Gender reference is women, test completion reference is test completion of TMT-B, occupation reference is being employed.

^1^ Partial eta squared derived from GLM including study as covariate

**Age of Onset**

| Parameter | Estimate | Std. Error | df | t | Sig. | 95% Confidence Interval | |  |
| --- | --- | --- | --- | --- | --- | --- | --- | --- |
|  |  |  |  |  |  | Lower Bound | Upper Bound | **Partial Eta Squared ^1^** |
| Intercept | -24.63 | 9.74 | 190.37 | -2.53 | 0.01 | -43.83 | -5.42 | 0.08 |
| age | 0.80 | 0.15 | 307.63 | 5.52 | <.001 | 0.52 | 1.09 | 0.00 |
| education | 0.27 | 0.28 | 239.68 | 0.97 | 0.33 | -0.28 | 0.82 | 0.01 |
| Gender [men] | -0.93 | 1.77 | 316.44 | -0.53 | 0.60 | -4.41 | 2.55 | 0.00 |
| Psychomotor speed (TMT A) | 0.00 | 0.03 | 316.59 | 0.13 | 0.90 | -0.05 | 0.06 | 0.28 |
| Occupation status [not employed] | -0.32 | 2.29 | 318.00 | -0.14 | 0.89 | -4.84 | 4.19 | 0.00 |
| Executive functioning (TMT-B test non-completer) | 1.84 | 3.11 | 311.82 | 0.59 | 0.55 | -4.28 | 7.96 | 0.00 |

Estimates of fixed effects derived from linear mixed models, random effect: study

Gender reference is women, test completion reference is test completion of TMT-B, occupation reference is being employed.

^1^ Partial eta squared derived from GLM including study as covariate

**Depression Severity**

| Parameter | Estimate | Std. Error | df | t | Sig. | 95% Confidence Interval | |  |
| --- | --- | --- | --- | --- | --- | --- | --- | --- |
|  |  |  |  |  |  | Lower Bound | Upper Bound | **Partial Eta Squared ^1^** |
| Intercept | 1.87 | 0.36 | 220.51 | 5.20 | <.001 | 1.16 | 2.58 | 0.09 |
| age | -0.01 | 0.00 | 346.16 | -2.82 | 0.01 | -0.02 | 0.00 | 0.00 |
| education | -0.02 | 0.01 | 347.67 | -1.51 | 0.13 | -0.03 | 0.00 | 0.01 |
| Gender [men] | -0.05 | 0.06 | 345.00 | -0.81 | 0.42 | -0.17 | 0.07 | 0.00 |
| Psychomotor speed (TMT A) | 0.00 | 0.00 | 344.31 | -1.41 | 0.16 | 0.00 | 0.00 | 0.30 |
| Occupation status [not employed] | 0.11 | 0.08 | 344.91 | 1.38 | 0.17 | -0.05 | 0.26 | 0.00 |
| Executive functioning (TMT-B test non-completer) | 0.14 | 0.11 | 345.14 | 1.29 | 0.20 | -0.07 | 0.36 | 0.01 |

Estimates of fixed effects derived from linear mixed models, random effect: study

Gender reference is women, test completion reference is test completion of TMT-B, occupation reference is being employed.

^1^ Partial eta squared derived from GLM including study as covariate

**Lithium Use**

| Parameter | Estimate | Std. Error | df | t | Sig. | 95% Confidence Interval | |  |
| --- | --- | --- | --- | --- | --- | --- | --- | --- |
|  |  |  |  |  |  | Lower Bound | Upper Bound | **Partial Eta Squared ^1^** |
| Intercept | 0.46 | 0.26 | 72.36 | 1.77 | 0.08 | -0.06 | 0.98 | 0.09 |
| age | 0.00 | 0.00 | 317.05 | -0.49 | 0.63 | -0.01 | 0.00 | 0.00 |
| education | 0.00 | 0.01 | 317.56 | -0.47 | 0.64 | -0.02 | 0.01 | 0.02 |
| Gender [men] | -0.08 | 0.04 | 316.89 | -1.88 | 0.06 | -0.16 | 0.00 | 0.00 |
| Psychomotor speed (TMT A) | 0.00 | 0.00 | 316.58 | 0.99 | 0.32 | 0.00 | 0.00 | 0.29 |
| Occupation status [not employed] | 0.01 | 0.05 | 316.65 | 0.16 | 0.87 | -0.09 | 0.11 | 0.00 |
| Executive functioning (TMT-B test non-completer) | -0.04 | 0.07 | 316.80 | -0.58 | 0.57 | -0.18 | 0.10 | 0.00 |

Estimates of fixed effects derived from linear mixed models, random effect: study

Gender reference is women, test completion reference is test completion of TMT-B, occupation reference is being employed.

^1^ Partial eta squared derived from GLM including study as covariate

**Anti-psychotics Use**

| Parameter | Estimate | Std. Error | df | t | Sig. | 95% Confidence Interval | |  |
| --- | --- | --- | --- | --- | --- | --- | --- | --- |
|  |  |  |  |  |  | Lower Bound | Upper Bound | **Partial Eta Squared ^1^** |
| Intercept | 0.75 | 0.30 | 250.27 | 2.50 | 0.01 | 0.16 | 1.35 | 0.09 |
| age | -0.01 | 0.00 | 316.00 | -1.41 | 0.16 | -0.01 | 0.00 | 0.00 |
| education | -0.01 | 0.01 | 315.95 | -1.52 | 0.13 | -0.03 | 0.00 | 0.02 |
| Gender [men] | -0.04 | 0.05 | 315.13 | -0.70 | 0.49 | -0.14 | 0.07 | 0.00 |
| Psychomotor speed (TMT A) | 0.00 | 0.00 | 313.96 | 1.30 | 0.20 | 0.00 | 0.00 | 0.29 |
| Occupation status [not employed] | 0.17 | 0.07 | 314.77 | 2.52 | 0.01 | 0.04 | 0.29 | 0.00 |
| Executive functioning (TMT-B test non-completer) | -0.02 | 0.09 | 315.36 | -0.21 | 0.83 | -0.20 | 0.16 | 0.00 |

Estimates of fixed effects derived from linear mixed models, random effect: study

Gender reference is women, test completion reference is test completion of TMT-B, occupation reference is being employed.

^1^ Partial eta squared derived from GLM including study as covariate

**H2e-M1a: Association of Executive Function with Functioning (GAF) in OABD TMT-B Test Completers**

| Parameter | Estimate | Std. Error | df | t | Sig. | 95% Confidence Interval | |  |
| --- | --- | --- | --- | --- | --- | --- | --- | --- |
|  |  |  |  |  |  | Lower Bound | Upper Bound | **Partial Eta Squared ^1^** |
| Intercept | -0.25 | 0.55 | 149.36 | -0.45 | 0.65 | -1.33 | 0.84 | 0.00 |
| age | 0.01 | 0.01 | 294.99 | 0.70 | 0.49 | -0.01 | 0.02 | 0.00 |
| education | -0.01 | 0.02 | 292.68 | -0.66 | 0.51 | -0.04 | 0.02 | 0.30 |
| Gender [men] | 0.01 | 0.09 | 293.16 | 0.08 | 0.94 | -0.17 | 0.18 | 0.00 |
| Antipsychotics [no current antipsychotic use] | 0.12 | 0.09 | 291.43 | 1.32 | 0.19 | -0.06 | 0.31 | 0.00 |
| Executive functioning (TMT-B) | -0.25 | 0.55 | 149.36 | -0.45 | 0.65 | -1.33 | 0.84 | 0.00 |

Estimates of fixed effects derived from linear mixed models, random effect: study

Gender reference is women. Antipsychotic reference is currently using antipsychotic medication.

^1^ Partial eta squared derived from GLM including study as covariate.

**H2e-M1b: Association of Executive Function with Functioning (GAF) in OABD TMT-B Test Completers**

| Parameter | Estimate | Std. Error | df | t | Sig. | 95% Confidence Interval | |  |
| --- | --- | --- | --- | --- | --- | --- | --- | --- |
|  |  |  |  |  |  | Lower Bound | Upper Bound | **Partial Eta Squared ^1^** |
| Intercept | -0.31 | 0.51 | 175.88 | -0.60 | 0.55 | -1.31 | 0.70 | 0.00 |
| age | 0.01 | 0.01 | 327.99 | 1.13 | 0.26 | -0.01 | 0.02 | 0.00 |
| education | -0.01 | 0.01 | 325.83 | -0.47 | 0.64 | -0.04 | 0.02 | 0.00 |
| Gender [men] | -0.02 | 0.08 | 325.54 | -0.18 | 0.86 | -0.18 | 0.15 | 0.00 |
| Executive functioning (TMT-B) | -0.20 | 0.05 | 323.30 | -4.09 | <.001 | -0.29 | -0.10 | 0.05 |

Estimates of fixed effects derived from linear mixed models, random effect: study

Gender reference is women.

^1^ Partial eta squared derived from GLM including study as covariate.

**H2e-M2: Association of Executive Function with Functioning (GAF) in OABD TMT-B Test Completers**

| Parameter | Estimate | Std. Error | df | t | Sig. | 95% Confidence Interval | |  |
| --- | --- | --- | --- | --- | --- | --- | --- | --- |
|  |  |  |  |  |  | Lower Bound | Upper Bound | **Partial Eta Squared ^1^** |
| Intercept | -0.43 | 0.54 | 59.14 | -0.79 | 0.43 | -1.52 | 0.66 | 0.00 |
| age | 0.01 | 0.01 | 290.97 | 1.18 | 0.24 | -0.01 | 0.02 | 0.01 |
| Gender [men] | -0.04 | 0.09 | 291.18 | -0.52 | 0.60 | -0.21 | 0.12 | 0.00 |
| Psychomotor speed (TMT-A) | -0.05 | 0.05 | 290.42 | -1.01 | 0.32 | -0.16 | 0.05 | 0.01 |
| Executive functioning (TMT-B) | -0.18 | 0.06 | 290.97 | -3.13 | 0.00 | -0.30 | -0.07 | 0.03 |

Estimates of fixed effects derived from linear mixed models, random effect: study

Gender reference is women.

^1^ Partial eta squared derived from GLM including study as covariate.

**H2e-M3: Association of Executive Function with Functioning (GAF) in OABD TMT-B Test Completers**

| Parameter | Estimate | Std. Error | df | t | Sig. | 95% Confidence Interval | |  |
| --- | --- | --- | --- | --- | --- | --- | --- | --- |
|  |  |  |  |  |  | Lower Bound | Upper Bound | **Partial Eta Squared ^1^** |
| Intercept | -0.25 | 0.51 | 181.25 | -0.48 | 0.63 | -1.25 | 0.76 | 0.00 |
| age | 0.01 | 0.01 | 318.98 | 1.55 | 0.12 | 0.00 | 0.03 | 0.01 |
| education | -0.01 | 0.01 | 317.08 | -0.71 | 0.48 | -0.04 | 0.02 | 0.00 |
| Gender [men] | 0.00 | 0.08 | 316.78 | -0.05 | 0.96 | -0.17 | 0.16 | 0.00 |
| Occupation status [not employed] | -0.28 | 0.10 | 315.07 | -2.78 | 0.01 | -0.48 | -0.08 | 0.02 |
| Executive functioning (TMT-B) | -0.25 | 0.51 | 181.25 | -0.48 | 0.63 | -1.25 | 0.76 | 0.00 |

Estimates of fixed effects derived from linear mixed models, random effect: study

Gender reference is women. Occupation reference is being employed.

^1^ Partial eta squared derived from GLM including study as covariate.

**H2e-M4: Association of Executive Function with Functioning (GAF) in OABD TMT-B Test Completers**

| Parameter | Estimate | Std. Error | df | t | Sig. | 95% Confidence Interval | |  |
| --- | --- | --- | --- | --- | --- | --- | --- | --- |
|  |  |  |  |  |  | Lower Bound | Upper Bound | **Partial Eta Squared ^1^** |
| Intercept | -0.44 | 0.55 | 65.15 | -0.80 | 0.43 | -1.54 | 0.66 | 0.00 |
| age | 0.01 | 0.01 | 285.96 | 1.40 | 0.16 | 0.00 | 0.02 | 0.01 |
| Gender [men] | -0.05 | 0.09 | 286.36 | -0.55 | 0.58 | -0.22 | 0.12 | 0.00 |
| Occupation status [not employed] | -0.13 | 0.11 | 286.02 | -1.21 | 0.23 | -0.35 | 0.08 | 0.01 |
| Psychomotor speed (TMT-A) | -0.04 | 0.05 | 285.57 | -0.80 | 0.42 | -0.15 | 0.06 | 0.01 |
| Executive functioning (TMT-B) | -0.44 | 0.55 | 65.15 | -0.80 | 0.43 | -1.54 | 0.66 | 0.00 |

Estimates of fixed effects derived from linear mixed models, random effect: study

Gender reference is women. Occupation reference is being employed.

^1^ Partial eta squared derived from GLM including study as covariate.

**H2f-M1a: Association of Executive Function with Global Cognition in OABD TMT-B Test Completers**

| Parameter | Estimate | Std. Error | df | t | Sig. | 95% Confidence Interval | |  |
| --- | --- | --- | --- | --- | --- | --- | --- | --- |
|  |  |  |  |  |  | Lower Bound | Upper Bound | **Partial Eta Squared ^1^** |
| Intercept | 0.45 | 0.42 | 113.26 | 1.09 | 0.28 | -0.37 | 1.28 | 0.00 |
| age | -0.02 | 0.01 | 396.90 | -3.02 | 0.00 | -0.03 | -0.01 | 0.03 |
| education | 0.05 | 0.01 | 395.26 | 3.83 | <.001 | 0.02 | 0.07 | 0.04 |
| Gender [men] | -0.21 | 0.07 | 393.72 | -3.13 | 0.00 | -0.35 | -0.08 | 0.03 |
| Antipsychotics [no current antipsychotic use] | 0.10 | 0.08 | 393.80 | 1.38 | 0.17 | -0.04 | 0.25 | 0.00 |
| Executive functioning (TMT-B) | -0.64 | 0.04 | 392.27 | -15.42 | <.001 | -0.72 | -0.56 | 0.38 |

Estimates of fixed effects derived from linear mixed models, random effect: study

Gender reference is women. Antipsychotic reference is currently using antipsychotic medication.

^1^ Partial eta squared derived from GLM including study as covariate.

**H2f-M1b: Association of Executive Function with Global Cognition in OABD TMT-B Test Completers**

| Parameter | Estimate | Std. Error | df | t | Sig. | 95% Confidence Interval | |  |
| --- | --- | --- | --- | --- | --- | --- | --- | --- |
|  |  |  |  |  |  | Lower Bound | Upper Bound | **Partial Eta Squared ^1^** |
| Intercept | 0.48 | 0.41 | 108.20 | 1.16 | 0.25 | -0.34 | 1.30 | 0.00 |
| age | -0.02 | 0.01 | 402.26 | -2.96 | 0.00 | -0.03 | -0.01 | 0.02 |
| education | 0.04 | 0.01 | 400.02 | 3.81 | <.001 | 0.02 | 0.07 | 0.04 |
| Gender [men] | -0.22 | 0.07 | 398.76 | -3.20 | 0.00 | -0.35 | -0.08 | 0.03 |
| Executive functioning (TMT-B) | -0.65 | 0.04 | 397.06 | -16.10 | <.001 | -0.73 | -0.57 | 0.40 |

Estimates of fixed effects derived from linear mixed models, random effect: study

Gender reference is women.

^1^ Partial eta squared derived from GLM including study as covariate.

**H2f-M2: Association of Executive Function with Global Cognition in OABD TMT-B Test Completers**

| Parameter | Estimate | Std. Error | df | t | Sig. | 95% Confidence Interval | |  |
| --- | --- | --- | --- | --- | --- | --- | --- | --- |
|  |  |  |  |  |  | Lower Bound | Upper Bound | **Partial Eta Squared ^1^** |
| Intercept | 0.79 | 0.34 | 231.09 | 2.32 | 0.02 | 0.12 | 1.45 | 0.03 |
| age | -0.01 | 0.00 | 370.57 | -1.53 | 0.13 | -0.02 | 0.00 | 0.01 |
| Gender [men] | -0.18 | 0.06 | 368.98 | -2.83 | 0.01 | -0.30 | -0.05 | 0.03 |
| Psychomotor speed (TMT-A) | -0.40 | 0.04 | 369.88 | -9.88 | <.001 | -0.48 | -0.32 | 0.23 |
| Executive functioning (TMT-B) | -0.47 | 0.05 | 368.30 | -10.36 | <.001 | -0.56 | -0.38 | 0.24 |

Estimates of fixed effects derived from linear mixed models, random effect: study

Gender reference is women.

^1^ Partial eta squared derived from GLM including study as covariate.

**H2f-M3: Association of Executive Function with Global Cognition in OABD TMT-B Test Completers**

| Parameter | Estimate | Std. Error | df | t | Sig. | 95% Confidence Interval | |  |
| --- | --- | --- | --- | --- | --- | --- | --- | --- |
|  |  |  |  |  |  | Lower Bound | Upper Bound | **Partial Eta Squared ^1^** |
| Intercept | 0.57 | 0.51 | 62.54 | 1.12 | 0.27 | -0.45 | 1.58 | 0.00 |
| age | -0.01 | 0.01 | 276.10 | -1.88 | 0.06 | -0.03 | 0.00 | 0.02 |
| education | 0.03 | 0.01 | 274.36 | 2.19 | 0.03 | 0.00 | 0.06 | 0.02 |
| Gender [men] | -0.24 | 0.08 | 274.38 | -3.06 | 0.00 | -0.39 | -0.09 | 0.04 |
| Occupation status [not employed] | -0.11 | 0.10 | 273.88 | -1.12 | 0.26 | -0.29 | 0.08 | 0.00 |
| Executive functioning (TMT-B) | 0.57 | 0.51 | 62.54 | 1.12 | 0.27 | -0.45 | 1.58 | 0.00 |

Estimates of fixed effects derived from linear mixed models, random effect: study

Gender reference is women. Occupation reference is being employed.

^1^ Partial eta squared derived from GLM including study as covariate.

**H2f-M4: Association of Executive Function with Global Cognition in OABD TMT-B Test Completers**

| Parameter | Estimate | Std. Error | df | t | Sig. | 95% Confidence Interval | |  |
| --- | --- | --- | --- | --- | --- | --- | --- | --- |
|  |  |  |  |  |  | Lower Bound | Upper Bound | **Partial Eta Squared ^1^** |
| Intercept | 0.65 | 0.39 | 179.23 | 1.67 | 0.10 | -0.12 | 1.41 | 0.04 |
| age | 0.00 | 0.01 | 250.42 | -0.69 | 0.49 | -0.02 | 0.01 | 0.00 |
| Gender [men] | -0.20 | 0.07 | 250.00 | -2.95 | 0.00 | -0.34 | -0.07 | 0.04 |
| Occupation status [not employed] | 0.05 | 0.09 | 249.81 | 0.55 | 0.58 | -0.12 | 0.22 | 0.00 |
| Psychomotor speed (TMT-A) | -0.39 | 0.04 | 250.02 | -9.21 | <.001 | -0.48 | -0.31 | 0.31 |
| Executive functioning (TMT-B) | 0.65 | 0.39 | 179.23 | 1.67 | 0.10 | -0.12 | 1.41 | 0.04 |

Estimates of fixed effects derived from linear mixed models, random effect: study

Gender reference is women. Occupation reference is being employed.

^1^ Partial eta squared derived from GLM including study as covariate.

**Supplementary Methods**

**Statistical analysis**

Specifically, the following analysis were conducted:

**1)** In the dataset of those who completed the TMT-B test, i.e. OABD and HC, linear mixed models controlling for study, age, education and gender, investigated executive function as dependent variable (DV) and diagnostic group as independent variable (IV) to compare executive function between diagnostic groups. Further, the moderating effect of age was explored using the interaction of diagnostic group * age.

**2)** Within the OABD patient group, while controlling for study, education and gender, linear mixed models were used with executive function (**a)** test completion vs non-completion within all OABD participants and **b)** test completion time within test completers) as DV and age as IV.

Further, while controlling for study, age, education and gender, linear mixed models were used with **b)** executive function as DV and clinical characteristics (i.e. depression severity, manic symptom severity, age of onset, current antipsychotic and lithium use) as IVs in separate models, False Discovery Rate (FDR)-correction was applied. Then, **c)** test completion category (completers vs non-completers) as DV and the above mentioned clinical variables were used to predict test completion versus non-completion.

Additional models included **d)** daily functioning or **e)** global cognition as DV, and executive function as IV. To these two linear mixed models, significant clinical characteristics as found in 2a) (i.e. current antipsychotic medication use) were added as additional independent variables of interest **(d) a**nd **e)).**
